# Evodiamine Augments NLRP3 Inflammasome Activation and Anti-bacterial Responses Through Inducing α-Tubulin Acetylation

**DOI:** 10.3389/fphar.2019.00290

**Published:** 2019-03-26

**Authors:** Chen-Guang Li, Qiong-Zhen Zeng, Ming-Ye Chen, Li-Hui Xu, Cheng-Cheng Zhang, Feng-Yi Mai, Chen-Ying Zeng, Xian-Hui He, Dong-Yun Ouyang

**Affiliations:** ^1^Department of Immunobiology, College of Life Science and Technology, Jinan University, Guangzhou, China; ^2^Department of Cell Biology, College of Life Science and Technology, Jinan University, Guangzhou, China

**Keywords:** evodiamine, acetylation, NLRP3 inflammasome, α-tubulin, pyroptosis

## Abstract

Evodiamine is a major ingredient of the plant *Evodia rutaecarpa*, which has long been used for treating infection-related diseases including diarrhea, beriberi and oral ulcer, but the underlying mechanism is unclear. Here we aimed to explore whether evodiamine influenced NLRP3 (NLR family, pyrin containing domain 3) inflammasome activation in macrophages, which is a critical mechanism for defending the host against pathogenic infections. We uncovered that evodiamine dose-dependently enhanced NLRP3 inflammasome activation in lipopolysaccharide-primed macrophages, as indicated by increased interleukin (IL)-1β production and caspase-1 cleavage, accompanied by increased ASC speck formation and pyroptosis. Mechanistically, evodiamine induced acetylation of α-tubulin around the microtubule organization center (indicated by γ-tubulin) in lipopolysaccharide-primed macrophages. Such evodiamine-mediated increases in NLRP3 activation and pyroptosis were attenuated by activators of α-tubulin deacetylase, resveratrol and NAD^+^, or dynein-specific inhibitor ciliobrevin A. Small interfering RNA knockdown of *αTAT1* (the gene encoding α-tubulin *N*-acetyltransferase) expression, which reduced α-tubulin acetylation, also diminished evodiamine-mediated augmentation of NLRP3 activation and pyroptosis. Evodiamine also enhanced NLRP3-mediated production of IL-1β and neutrophil recruitment *in vivo*. Moreover, evodiamine administration evidently improved survival of mice with lethal bacterial infection, accompanied by increased production of IL-1β and interferon-γ, decreased bacterial load, and dampened liver inflammation. Resveratrol treatment reversed evodiamine-induced increases of IL-1β and interferon-γ, and decreased bacterial clearance in mice. Collectively, our results indicated that evodiamine augmented the NLRP3 inflammasome activation through inducing α-tubulin acetylation, thereby conferring intensified innate immunity against bacterial infection.

## Introduction

Macrophages are critical innate immune cells in tissues to sense pathogenic infections or tissue damages through multiple cytosolic pattern recognition receptors (PRRs) leading to the formation of inflammasomes, which are multimeric protein complexes that are induced in the cytosol as a platform for the activation of caspase-1 (Broz and Dixit, 2016). Several types of inflammasomes including AIM2, NLRP1, NLRP3 (NLR family, pyrin containing domain 3), NLRC4, and pyrin inflammasomes, have been reported (Broz and Dixit, 2016). Among them, the NLRP3 inflammasome is the most extensively investigated one (Jo et al., 2016). It is believed that NLRP3 inflammasome activation requires two signals (Jo et al., 2016). The first is the priming signal, which is provided by binding of pathogen-associated molecular patterns (PAMPs) to PRRs, culminating in the expression of critical components of inflammasomes including NLRP3, pro-interleukin (IL)-1β, and pro-IL-18. The second signal is provided by a broad spectrum of stimuli including extracellular ATP, pore-forming toxins (e.g., nigericin), particulates (e.g., uric acid crystals, silica, and alum), and pathogens (bacteria, fungi, protozoan, and virus) (Martinon et al., 2006; Iwasaki and Medzhitov, 2015; Man and Kanneganti, 2015). Upon such second stimulation, NLRP3 recruits the adaptor protein ASC (apoptosis-associated speck-like protein containing a caspase recruitment domain), which in turn interacts with pro-caspase-1, leading to the formation of NLRP3 inflammasome. The assembly of NLRP3 inflammasome triggers the autocatalytic cleavage and activation of caspase-1 (Jo et al., 2016). Biologically active caspase-1 then processes pro-IL-1β and pro-IL-18 into their mature forms, which can be released from the cell (Jo et al., 2016). NLRP3 inflammasome activation is therefore regarded as a critical host defense mechanism against pathogenic infections.

As an infection/damage-sensing protein, NLRP3 can be regulated by many post-translational modifications including ubiquitination (Song et al., 2016) and phosphorylation (Guo et al., 2016, 2017). Its activation pathway is also regulated by many regulatory proteins (Bryant and Fitzgerald, 2009; He et al., 2016; Jo et al., 2016). Interestingly, recent studies have showed that microtubules play important roles in regulating NLRP3 inflammasome activation. For example, the trafficking of NLRP3 inflammasome components and their assembly rely on the microtubule-mediated transport machinery within macrophages (Misawa et al., 2013; Li et al., 2017c). It has been demonstrated that α-tubulin acetylation increases the flexibility of microtubules and accelerates the movement of motor molecules (Balabanian et al., 2017). Importantly, acetylated α-tubulin promotes dynein-mediated trafficking of mitochondria (a cargo of NLRP3 inflammasome adapter ASC) along microtubules to the minus end (i.e., the perinuclear region), thus enhancing the apposition of ASC with NLRP3 on the endoplasmic reticulum (ER) and the assembly of the NLRP3 inflammasome (Misawa et al., 2013). α-Tubulin in microtubules can be acetylated by α-tubulin N-acetyltransferase (αTAT1/MEC-17), and deacetylated by NAD^+^-dependent α-tubulin deacetylase sirtuin 2 (SIRT2) or histone deacetylase 6 (HDAC6) (Hubbert et al., 2002; Akella et al., 2010; Kalebic et al., 2013), though HDAC6 is not involved in NLRP3 inflammasome activation (Misawa et al., 2013). Consistent with these reports, NLRP3 inflammasome inducers (such as ATP and nigericin) induce α-tubulin acetylation by inactivating SIRT2, whereas SIRT2 activators resveratrol and NAD^+^ suppress α-tubulin acetylation, thus inhibiting ASC-mediated NLRP3 inflammasome assembly and activation (Misawa et al., 2013, 2015). Therefore, microtubules act as tracks for the rapid subcellular transport of ASC and other NLRP3 inflammasome components, and α-tubulin acetylation may affect their trafficking thus controlling NLRP3 inflammasome activation, highlighting the microtubule cytoskeleton as an important target for discovering drugs to regulate NLRP3 activation.

Evodiamine is an indoloquinazoline alkaloid isolated from the fruits of *Evodia rutaecarpa* (Chinese name: Wu-Zhu-Yu), a plant belonging to the family of Rutaceae (Shoji et al., 1986). Evodia fruits have been used in traditional Chinese and Japanese medicine for the treatment of various infection-related diseases including diarrhea, beriberi, and oral ulcer (Liao et al., 2011). Recent studies have shown that evodiamine possesses a variety of pharmacological activities. For example, evodiamine has been reported to inhibit hypoxia-induced inflammatory responses in murine macrophages via suppressing the activation of hypoxia inducible factor-α (Liu et al., 2009). A more recent study using interactomics approach has identified ABC transporter A1 (ABCA1) as the direct target of evodiamine (Wang et al., 2018). Mechanistic studies showed that evodiamine directly bound ABCA1, and increased the stability of ABCA1 protein, thereby promoting cholesterol efflux from human macrophages (Wang et al., 2018). Evodiamine may also be an agonist of TRPV1 (Pearce et al., 2004). Moreover, evodiamine prevents platelet-derived growth factor-induced migration of vascular smooth muscle cells by activating PPAR-γ (Ge et al., 2015). It also ameliorates liver and cardiac fibrosis (Jiang et al., 2017; Yang D. et al., 2018) as well as colitis (Shen et al., 2019). Besides, evodiamine has been reported to have anti-tumor activities: it induces apoptosis in many kinds of tumor cells, including hepatic carcinoma (Qiu et al., 2016), lung cancer (Mohan et al., 2016), colorectal cancer (Zhou et al., 2019), osteosarcoma (Meng et al., 2015), and glioma (Wu et al., 2017), thus preventing their proliferation and migration.

Interestingly, it has been shown that evodiamine can target microtubules by increasing tubulin polymerization or by inhibiting microtubule polymerization in a variety of human cancer cells (Huang et al., 2004, 2005; Liao et al., 2005). As microtubules play important roles in mediating NLRP3 inflammasome activation (Misawa et al., 2013; Li et al., 2017c), it is of great interest to know whether and how the microtubule-targeting agent evodiamine affects the activation of the NLRP3 inflammasome in macrophages. We found in the present study that evodiamine was able to enhance NLRP3 inflammasome activation by promoting the accumulation of acetylated α-tubulin in macrophages. Moreover, evodiamine administration markedly augmented the innate immune responses in a mouse model of bacterial infection thereby enhancing bacterial clearance and improving animal survival. Our results highlight evodiamine as a novel agent for promoting NLRP3 inflammasome activation to intensify antibacterial responses.

## Materials and Methods

### Reagents and Antibodies

**FIGURE 1:**
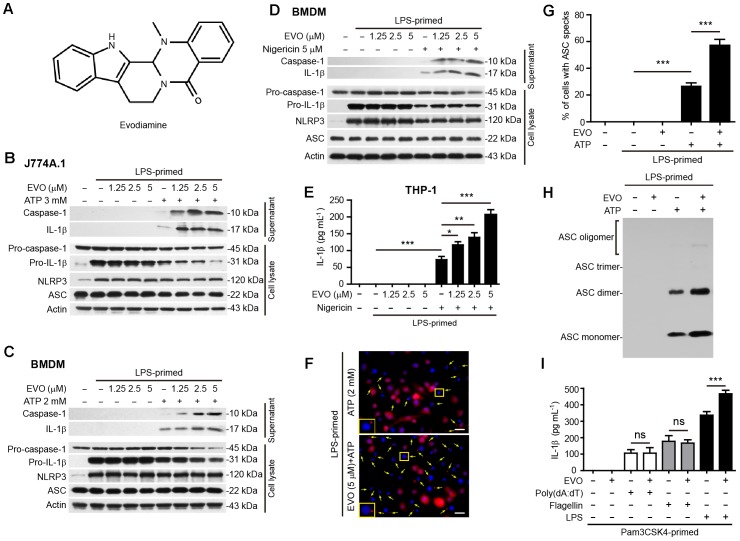
Evodiamine specifically promoted NLRP3 inflammasome activation in macrophages. **(A)** The chemical strcuture of evodiamine. **(B–D)** J774A.1 cells **(B)** and bone marrow-derived macrophages (BMDMs) **(C,D)** were treated as described in Section “*Materials and Methods*.” Western blotting was used to assess the expression levels of indicated proteins in the cell lysates and culture supernatants, respectively. Actin was used as a loading control for cell lysates. **(E)** Human THP-1 macrophages were induced by PMA (500 nM) treatment for 16 h. The cells were then primed with LPS (1 μg/ml) for 4 h, and pre-treated with series concentrations of evodiamine for 1 h, followed by incubation with nigericin (20 μM) for 1 h in the absence of LPS. The levels of soluble IL-1β in the culture supernatants were detected by cytometric bead array (CBA) assay. Data are shown as mean ± SD (*n* = 5). One-way analysis of variance (ANOVA): *P* < 0.0001; Tukey’s *post hoc* test: ^∗^*P* < 0.05, ^∗∗^*P* < 0.01, ^∗∗∗^*P* < 0.001. **(F)** BMDMs were treated as in **(C)**. Representative immunofluorescence images showing ASC (red) subcellular distribution. Nuclei (blue) were revealed by Hoechst 33342. Yellow arrows indicate ASC specks and the enlarged inset showing cells with an ASC speck. Scale bars, 20 μm. **(G)** Percentages of cells with an ASC speck relative to the total number of cells from 5 random fields (one field per well) each containing ∼50 cells. Data are shown as mean ± SD (*n* = 5). One-way ANOVA: *P* < 0.0001; Tukey’s *post hoc* test: ^∗∗∗^*P* < 0.001. **(H)** Western blot analysis for ASC in Triton-X 100 insoluble cytosolic pellets cross-linked with disuccinimidyl suberate. **(I)** J774A.1 cells were primed with Pam3CSK4 (1 μg/ml) for 4 h, then pre-treated with evodiamine 1 h, and followed by transfection with 2 μg/ml Poly(dA:dT), 0.5 μg/ml flagellin or 2.5 μg/ml LPS for 16 h, respectively. Soluble IL-1β levels in the culture supernatants was quantified by CBA assay. Data are shown as mean ± SD (*n* = 5). The experiments were performed three times independently. ^∗∗∗^*P* < 0.001, ns, not significant, by two-tailed Student’s *t*-test. EVO, evodiamine; Pam, Pam3CSK4.

Evodiamine (E101966; purity ≥ 99%; formula: C_19_H_17_N_3_O; formula weight: 303.36; structure: see Figure 1A) was purchased from Aladdin (Shanghai, China), dissolved in DMSO at 50 mM and stored at -20°C. Ciliobrevin A (S8249) was obtained from Selleck (Houston, TX, United States). Resveratrol (R5010), NAD^+^ (β-nicotinamide adenine dinucleotide hydrate) (N7004), ATP (A6419), lipopolysaccharide (LPS) (*Escherichia coli* O111:B4) (L4391), disuccinimidyl suberate (S1885), Hoechst 33342 (B2261), propidium iodide (PI) (P4170), anti-γ-tubulin (T5326), dimethyl sulfoxide (DMSO) (D8418), Tween-80 (P8074) and Tween-20 (P1379) were bought from Sigma-Aldrich (St. Louis, MO, United States). NAD^+^/NADH assay kit with WST-8 (S0175), Phorbol-12-myristate-13-acetate (PMA) (S1819), cell lysis buffer (P0013) and phenylmethanesulfonyl fluoride (PMSF) (ST505) were obtained from Beyotime (Shanghai, China). Nigericin (#tlrl-nig), monosodium urate crystal (MSU) (#tlrl-msu), Pam3CSK4 (#tlrl-pms), Poly(dA:dT) (#tlrl-patn) and FLA-PA Ultrapure (purified flagellin from *Pseudomonas aeruginosa*) (#tlrl-pafla) were obtained from InvivoGen (San Diego, CA, United States). Lipofectamine 2000 (11668-030), Lipofectamine RNAiMAX (13778-075), Dulbecco’s Modified Eagle’s Medium (DMEM) medium with high glucose, RPMI-1640, Opti-MEM, fetal bovine serum (FBS), streptomycin and penicillin were products of Thermo Fisher/Invitrogen (Carlsbad, CA, United States). FuGENE HD transfection reagent (E2311) was from Promega (Madison, WI, United States). The anti-NLRP3 antibody (AG-20B-0014) was purchased from Adipogen AG (Liestal, Switzerland). The antibody against actin (sc-1616-R) was purchased from Santa Cruz Biotechnology (Dallas, TX, United States). Specific antibodies against IL-1β (#12242), ASC (#67824), ASC-AlexaFluor488 (#17507), α-tubulin (#3873), acetyl(lysine40)-α-tubulin (#5335), horse-radish peroxidase (HRP)-linked horse anti-mouse IgG (#7076) and HRP-linked goat anti-rabbit IgG (#7074) were products of Cell Signaling Technology (Danvers, MA, United States). The antibodies against pro-caspase1+p10+p12 (ab179515), GSDMD (ab209845), αTAT1 (MEC-17) (ab58742), TOM20 (ab75985), and anti-Armenian hamster IgG H&L (HRP) (ab5745) were purchased from Abcam (Cambridge, United Kingdom). CF568 goat-anti-rabbit IgG (H+L), highly cross-adsorbed (#20103) and CF488A-conjugated goat-anti-mouse IgG, highly cross-adsorbed (#20018) were obtained from Biotium (Hayward, CA, United States). Anti-mouse CD11b FITC (#11-0112) and anti-mouse Ly-6G PE (#12-9668) were obtained from eBioscience (San Diego, CA, United States).

### Animals

Female C57BL/6 mice (6–8 weeks of age with weight between 18–20 g) were bought from the Experimental Animal Center of Southern Medical University (Guangzhou, China). All animals were acclimatized for 1 week before experiments and maintained under a strict 12 h/12 h dark/light cycle condition, with free access to water and food. Mice were divided randomly into different groups and the investigators were not blind to the experimental groups. All animal experiments were performed in accordance with the institutional guidelines for the care and use of animals of Jinan University. The protocol was approved by the Committee on the Ethics of Animal Experiments of Jinan University (JNU20170305).

### Cell Culture and Differentiation

Mouse macrophage cell line J774A.1 was obtained from the Kunming Cell Bank of Type Culture Collection Chinese Academy of Sciences (Kunming, China). The cells were maintained in complete DMEM medium (containing 10% FBS, 100 IU/ml penicillin, 100 μg/ml streptomycin, and 2 mM L-glutamine) and cultured at 37°C in a humidified incubator with 5% CO_2_. The cells were sub-cultured every 2–3 days by using a cell scraper to split cells. THP-1 cells (kindly provided by Dr. Yao Wang of Sun Yat-sen University, Guangzhou) were cultured in RPMI-1640 supplemented with 10% FBS and 50 μM β-mercaptoethanol. THP-1 cells were differentiated into macrophages by adding 500 nM of PMA for 16 h. Bone marrow-derived macrophages (BMDMs) from mice were harvested and differentiated as previously described (Kayagaki et al., 2011; Li et al., 2017b). Briefly, mice were sacrificed and bone marrow cells were collected from the femurs. Bone marrow cells were re-suspended in BM-Mac medium (80% DMEM medium containing 10% FBS plus 20% M-CSF-conditioned medium from L929 cells). Subsequently, the cells were seeded in 10-cm petri dishes with 10 ml BM-Mac medium and cultured at 37°C in a humidified incubator of 5% CO_2_. BMDMs were ready for experiments after 6 days.

### Inflammasome Activation

J774A.1 cells were seeded in 24-well plates at 8 × 10^4^ cells/well or in 6-well plates at 3 × 10^5^ cells/well, and BMDMs were cultured in 24-well plates at 1.5 × 10^5^ cells/well or in 6-well plates at 1.2 × 10^6^ cells/well, and cultured at 37°C overnight. Before inflammasome activation, cells were primed in Opti-MEM with 500 ng/ml LPS for 4 h, and then the cells were pre-treated with the following reagents: 5 μM of resveratrol for 30 min; 10 μM of NAD^+^ for 30 min; or 30 μM of ciliobrevin A for 30 min before incubation with indicated doses of evodiamine for 1 h. Canonical NLRP3 inflammasome activation was induced by the following treatment as indicated: 3 mM ATP for 1 h in J774A.1 cells; 2 mM ATP for 30 min or 5 μM of nigericin for 1 h in BMDMs; 20 μM of nigericin for 2 h in THP-1 cells. For non-canonical inflammasome activation, cells were primed with 1 μg/ml Pam3CSK4 for 4 h, and after that the medium was replaced with 2.5 μg/ml LPS plus 0.25% v/v FuGENE HD for 16 h. To trigger the activation of NLRC4 and AIM2 inflammasome, the cells were primed with 1 μg/ml Pam3CSK4 for 4 h, and then transfected with 0.5 μg/ml flagellin plus 0.25% v/v FuGENE HD for 16 h; or 2 μg/ml Poly(dA:dT) plus 0.1% v/v Lipofectamine 2000 for 16 h. Supernatants were collected for analyzing IL-1β by cytometric bead array (CBA).

### Cell Death Assay

Cell death was measured by PI incorporation as described previously (Py et al., 2014; Li et al., 2017b). Cells were cultured in 24-well plates and primed with 500 ng/ml LPS in Opti-MEM for 4 h. Subsequently, cells were treated with indicated concentrations of evodiamine in Opti-MEM for 1 h followed by stimulation with ATP or nigericin for indicated time periods. The cells were stained with PI solution (2 μg/ml PI plus 5 μg/ml Hoechst 33342) for 10 min at room temperature and observed immediately by live imaging using Zeiss Axio Observer D1 microscope equipped with a Zeiss LD Plan-Neofluar 20 × /0.4 Korr M27 objective lens (Carl Zeiss MicroImaging GmbH, Göttingen, Germany). Fluorescence images were captured with a Zeiss AxioCam MR R3 cooled CCD camera controlled with ZEN software (Carl Zeiss).

### Immunofluorescence Microscopy

Immunofluorescence analysis was performed as previously described (Zha et al., 2016; Li et al., 2017c). In brief, cells were seeded in glass-bottomed dishes (1 × 10^5^ cells/dish) and cultured at 37°C overnight. Cells were primed with 500 ng/ml LPS in Opti-MEM for 4 h. Then the cells were treated with evodiamine for 1 h, followed by treatment with ATP or nigericin for indicated time periods in Opti-MEM. After fixation, permeabilization and blocking, the cells were incubated with primary antibodies, followed by staining with CF568-conjugated goat-anti-rabbit IgG and CF488A-conjugated goat-anti-mouse IgG. After staining with Hoechst 33342 solution (5 μg/ml in PBS) to reveal the nuclei, the cells were observed under a Zeiss Axio Observer D1 microscope with a Zeiss LD Plan-Neofluar 40 × /0.6 Korr M27 objective lens or with a Zeiss LD Plan-Neofluar 100 × /0.6 Korr M27 objective lens (Carl Zeiss MicroImaging GmbH, Göttingen, Germany). Fluorescence images were captured by a Zeiss AxioCam MR R3 cooled CCD camera controlled with ZEN software (Carl Zeiss).

### Precipitation of Soluble Proteins in Supernatants

Soluble proteins in culture supernatants were precipitated as previously described (Kayagaki et al., 2011; Liu et al., 2017). The precipitated proteins were dissolved in equal volume of 2× sodium dodecylsulfate-polyacrylamide gel electrophoresis (SDS-PAGE) sample loading buffer and subjected to Western blot analysis of mature IL-1β and caspase-1p10.

### Western Blot Analysis

Western blotting was performed essentially as previously described (Li et al., 2017a). Briefly, total proteins were separated by SDS-PAGE and electro-transferred to PVDF membranes (#03010040001; Roche Diagnostics GmbH, Mannheim, Germany). The membranes were blocked by blocking buffer (PBS containing 3% FBS and 0.1% Tween-20) for 1 h and incubated with indicated primary antibody overnight at 4°C, followed by incubation with appropriate HRP-linked secondary antibody (horse anti-mouse or goat anti-rabbit IgG). Bands were revealed with an enhanced chemiluminescence kit (BeyoECL Plus; Beyotime, Shanghai, China) and recorded by X-ray films (Carestream, Xiamen, China). The blot images were captured by FluorChem8000 imaging system (AlphaInnotech, San Leandro, CA, United States). The gray values were analyzed by AlphaEaseFC 4.0 (AlphaInnotech).

### ASC Oligomer Cross-Linking

To determine the formation of ASC oligomer, chemical cross-linking assay using disuccinimidyl suberate (DSS) was performed as described previously (He et al., 2014; Coll et al., 2015). Briefly, BMDMs were seeded in 6-well plates at 1 × 10^6^ cells/well. After appropriate treatments, cells were lysed with cold PBS containing 0.5% Triton X-100, and the cell lysates were centrifuged at 6000 × *g* for 15 min at 4°C. The Triton X-100 insoluble pellets were washed twice with PBS and then re-suspended in 200 ml PBS. Freshly prepared disuccinimidyl suberate (2 mM) was added to the re-suspended pellets and the suspension was incubated at room temperature for 30 min with rotation. The cross-linked pellets were collected by centrifugation at 6000 × *g* for 15 min at 4°C and re-dissolved in 25 μl of 2× SDS-PAGE sample loading buffer. Samples were boiled for 5 min and subjected to Western blot analysis.

### Small Interfering RNA (siRNA)

The siRNA (5′-GGA TAC AAG AAG CTC TTT G-3′) duplexes targeting mouse *αTAT1* (*αTAT1* siRNA) was based on published study (Misawa et al., 2013) and negative control (NC) siRNA was designed and synthesized by RiboBio (Guangzhou, China). The siRNA transfection was performed using transfection reagent Lipofectamine RNAiMAX (Invitrogen) according to the instruction provided by the manufacturers. Briefly, the siRNA was added to each well at a final concentration of 100 nM. Six hours later, media was replaced with DMEM containing 10% FBS and the cells were incubated for 72 h. αTAT1 expression levels were determined by using Western blotting.

### NAD^+^ Measurement

After appropriate treatments, J774A.1 cells (1 × 10^6^ cells/sample) were collected and intracellular NAD^+^ levels were determined by using NAD^+^/NADH assay kit with WST-8 according to the manufacturer’s instructions. In brief, cells were lysed with 200 μl of cold lysis buffer. To measure total NAD^+^/NADH, 20 μl of cell lysates was added to a 96-well plate. To measure NADH, the lysed cell suspension was incubated at 60°C for 30 min and 20 μl was added to a 96-well plate. Subsequently, 90 μl of alcohol dehydrogenase was added and incubated at 37°C for 10 min. Finally, 10 μl of chromogenic solution was added to the plate and the mixture was incubated at 37°C for 30 min. Standard curve was generated and measured at the same time as the samples. The absorbance values were measured at 450 nm and analyzed on a plate reader (Multiskan FC; Thermo Fisher Scientific, Carlsbad, CA, United States). The amount of NAD^+^ was derived by subtracting NADH from total NAD^+^/NADH.

### MSU-Induced Mouse Peritonitis

Mouse intraperitoneally injected with MSU is a well-established peritonitis animal model that has been described previously (Martinon et al., 2006). Eight-week-old mice were randomly assigned into two independent groups (group size: *n* = 5): MSU group and MSU + evodiamine group. They were administered intragastrically (i.g.) with evodiamine (20 mg/kg body weight) or vehicle (2% Tween-80 in PBS) for 1 h, respectively, before intraperitoneally injected with MSU in 0.5 ml sterile PBS (1 mg per mouse). After 6 h, the mice were sacrificed by cervical dislocation and the peritoneal cavity were lavaged with 1.5 ml cold PBS. IL-1β in the peritoneal lavage fluids was determined by using CBA. Neutrophils in the peritoneal exudate cells were stained with anti-mouse CD11b FITC and anti-mouse Ly-6G PE and analyzed by flow cytometry.

### Mouse Model of Bacterial Infection

The mouse model of bacterial infection was established with reference to previous reports (Chung et al., 2008; Wegiel et al., 2014). In brief, *E. coli* (DH5α strain) was grown in lysogeny broth (LB) media at 37°C overnight, and then re-inoculated into fresh LB media and grown for 4 h at 37°C. The viable bacteria were collected by centrifugation at 2 600 × *g* for 10 min, washed with PBS, and then re-suspended in appropriate volume of PBS. Mice were randomly divided into three groups (group size: *n* = 10), which were administered intragastrically (i.g.) once with evodiamine solution (10 or 20 mg/kg body weight) or vehicle (2% Tween-80 in PBS), respectively. Three hours later, viable *E. coli* cells (2 × 10^9^ CFU/mouse) in 0.5 mL of PBS were injected into the peritoneal cavity of each mouse, and 1 h later, they were administered (i.g.) once again with evodiamine solution or vehicle, respectively. Mouse survival was monitored every 6 h for five consecutive days. In a parallel experiment, mice were treated similarly and were anesthetized by sodium pentobarbital (40 mg/kg; i.p.) for blood collection at 4 and 8 h post bacterial infection. The bacterial loads were measured as described previously (Pan et al., 2015). Their anti-coagulated peripheral blood and sera were collected, respectively, and cytokine levels in the sera were measure by CBA. For blocking the *in vivo* effects of evodiamine, mice were administered (i.g.) with resveratrol solution (50 or 100 mg/kg body weight) or vehicle (2% Tween-80 in PBS). After 30 min, mice were gavaged with evodiamine solution (20 mg/kg body weight) or vehicle, respectively. Three hours later, viable *E. coli* cells (1 × 10^9^ CFU/mouse) in 0.5 ml of PBS were injected into the peritoneal cavity of each mouse. The mice were administered (i.g.) once again with evodiamine solution or vehicle 1 h after bacterial infection. Eight hours later, the mice were anesthetized by sodium pentobarbital (40 mg/kg; i.p.) and their anti-coagulated peripheral blood and sera were collected, respectively, and cytokines levels were measured by CBA. The livers were removed for histopathological analysis.

### Histopathology

Mice were sacrificed 8 h after bacterial infection. The livers were isolated and fixed in 4% neutral formaldehyde and embedded in paraffin. Paraffin slices of the tissues were stained with hematoxylin and eosin. Images were captured under the Zeiss Axio Observer D1 microscope armed with a color CCD (Zeiss).

### Flow Cytometry

For phenotyping analysis, anti-coagulated peripheral blood cells were collected and washed with PBS-F (PBS containing 0.1% NaN_3_ and 3% FBS), followed by staining with FITC-labeled anti-mouse CD11b and PE-labeled anti-mouse Ly-6G at 4°C for 30 min. Red blood cells were lysed. After washing with PBS-F, cells were fixed with 4% paraformaldehyde in PBS and then analyzed on a flow cytometer (Attune NxT acoustic focusing cytometer; Thermo Fisher Scientific, Carlsbad, CA, United States). Data were acquired and analyzed by using the Attune NxT software (Thermo Fisher Scientific).

### Detection of Soluble Cytokines

Soluble cytokines in mouse sera and in cell culture supernatants were collected and measured by cytometric bead array (CBA) mouse IL-1β Flex Set (#558266), mouse inflammation kit (#552364) and human inflammatory cytokines kit (#551811) (BD Biosciences, San Jose, CA, United States) according to the manufacturer’s instructions. Briefly, 50 μl of each standard mix and serum samples were added to flow cytometric tubes, respectively, followed by adding 50 μl of phycoerythrin detection reagent. The mixtures were incubated at room temperature for 2 h. After washing, the bead pellets were resuspended in 300 μl of wash buffer and analyzed on a flow cytometer (Attune NxT acoustic focusing cytometer). Data were acquired and analyzed by using the Attune NxT software (Thermo Fisher Scientific).

### Statistical Analysis

All experiments were performed three times independently. The data were expressed as mean ± standard deviation (SD), and analyzed for statistical significance using GraphPad Prism 7.0 (GraphPad Software Inc, San Diego, CA, United States). One-way analysis of variance (ANOVA) followed by Tukey *post hoc* test was used to analyze the statistical significance among multiple groups. Two-tailed Student’s *t-*test was performed to analyze the statistical significance between two groups. Kaplan–Meier survival curves were adopted for analysis of mouse survival, and the log-rank (Mantel-Cox) test was used to assess for differences in survival. *P*-values < 0.05 were considered statistically significant.

## Results

### NLRP3 Inflammasome Activation in Macrophages Is Specifically Augmented by Evodiamine Pre-treatment

Although it has been reported that evodiamine can target microtubules (Huang et al., 2004, 2005; Liao et al., 2005), it is still unknown whether this phytochemical affects inflammasome activation. To address this issue, we initially assessed the influence of evodiamine on the activation of the canonical NLRP3 inflammasome in LPS-primed mouse macrophage J774A.1 cell line and BMDMs, using ATP or nigericin as second stimulators. Western blotting showed that LPS priming induced the expression of NLRP3 and pro-IL-1β proteins, whereas pro-caspase-1 and ASC were constitutively expressed in macrophages irrespective of LPS priming, which is consistent with previous studies (Kayagaki et al., 2013). Upon ATP or nigericin triggering, both caspase-1p10 (the cleaved form of caspase-1; 10 kDa) and mature IL-1β (17 kDa) were released into the culture supernatants. Notably, evodiamine pre-treatment dose-dependently increased the release of caspase-1p10 and mature IL-1β, indicative of enhanced activation of the NLRP3 inflammasome. Without ATP or nigericin triggering, however, evodiamine alone did not induce the release of these contents into supernatants (Figure 1B–D and Supplementary Figure S1). Similarly, evodiamine dose-dependently increased nigericin-induced IL-1β secretion in the supernatants of THP-1-derivied human macrophages, as assayed by CBA (Figure 1E). Immunofluorescence microscopy revealed that evodiamine significantly increased the formation of ASC specks (one in each cell) in the cytosol (Figure 1F,G), which is another marker of NLRP3 inflammasome activation. Consistent with the increase of ASC specks by evodiamine, ATP-induced formation of ASC oligomers was markedly increased by evodiamine, as detected by ASC cross-linking assay (Figure 1H). Together, these results indicated that evodiamine promoted NLRP3 inflammasome activation probably by enhancing ASC polymerization and the inflammasome assembly in both murine and human macrophages.

We next explored whether it could affect the activation of non-canonical NLRP3 inflammasome or other inflammasomes including NLRC4 and AIM2 inflammasomes. NLRC4, AIM2, and non-canonical NLRP3 inflammasome activation was induced by transfecting flagellin, poly(dA:dT), and LPS, respectively, into Pam3CSK4-primed macrophages. The results showed that evodiamine pre-treatment increased IL-1β secretion upon LPS transfecting, indicative of enhanced non-canonical NLRP3 inflammasome activation (Figure 1I). However, evodiamine did not influence the secretion of IL-1β in response to flagellin or poly(dA:dT) transfection (Figure 1I). These results demonstrated that evodiamine promoted NLRP3, but not NLRC4 and AIM2, inflammasome activation. All together, these results demonstrated that evodiamine specifically augmented NLRP3 inflammasome activation in both mouse and human macrophages.

### NLRP3-Mediated Pyroptosis Is Enhanced in Macrophages Treated With Evodiamine

**FIGURE 2:**
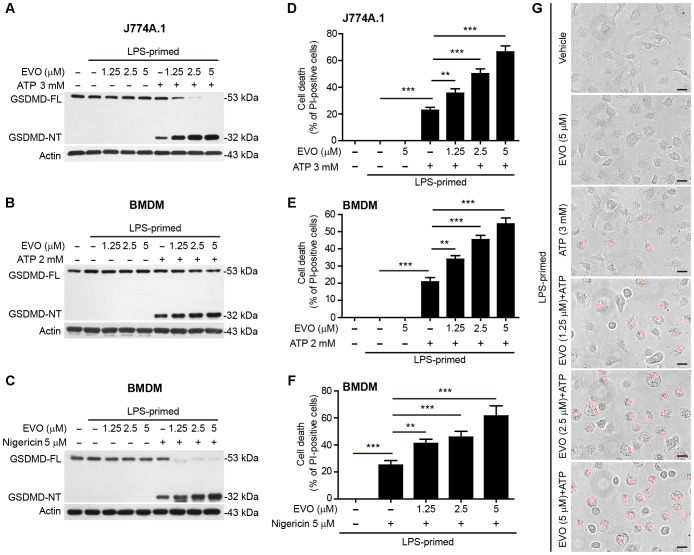
Evodiamine promoted ATP- or nigericin-induced pyroptosis in LPS-primed macrophages. J774A.1 cells **(A,D)** were treated as in Figure 1B. BMDMs in **(B,E,G)** were treated as in Figure 1C, and other BMDMs **(C,F)** were treated as in Figure 1D. **(A–C)** Western blotting was used to assess the expression levels of indicated proteins in the cell lysates. Actin was used as a loading control for cell lysates. **(D–G)** Cell death was measured by staining with propidium iodide (PI) (red, staining dead cells) for 10 min. The images were captured by fluorescence microscopy, the merged images show PI fluorescence with bright-field images. One set of representative images of three independent experiments are shown **(G)**. Scale bars, 20 μm. PI-positive cells were quantified by counting 5 randomly chosen fields (one field per well) containing around 100 cells each. Data are shown as mean ± SD (*n* = 5). One-way analysis of variance (ANOVA): *P* < 0.0001 **(D–F)**; Tukey *post hoc* test: ^∗∗^*P* < 0.01, ^∗∗∗^*P* < 0.001. GSDMD-FL, full-length GSDMD; GSDMD-NT, GSDMD N-terminal fragment; EVO, evodiamine.

NLRP3 inflammasome provides a platform for the autocatalytic activation of caspase-1, which not only causes IL-1β maturation but also cleaves gasdermin D (GSDMD) to generate its N-terminal fragment (GSDMD-NT) to mediate pyroptosis—a rapid proinflammatory programmed cell death (Shi et al., 2015). Western blot analysis showed that evodiamine markedly increased GSDMD-NT levels in ATP-treated J774A.1 cells (Figure 2A) and in ATP- or nigericin-treated BMDMs (Figure 2B,C). In agreement with the pore-forming activity of GSDMD-NT in the plasma membrane that can be assessed by PI incorporation (He et al., 2015), evodiamine pre-treatment significantly increased lytic cell death in a dose-dependent manner in ATP- or nigericin-treated macrophages (Figure 2D–F). Morphologically, evodiamine-enhanced cell death was similar to ATP- or nigericin-induced pyroptosis with rapid cellular swelling, but not membrane blebbing observed in apoptosis (Figure 2G). Consistent with the results that evodiamine failed to influence IL-1β release upon the stimuli for NLRC4 and AIM2 inflammasome activation as mentioned above (Figure 1I), evodiamine pre-treatment did not affect pyroptosis in these cells (Supplementary Figures S2A,B). However, evodiamine markedly enhanced non-canonical NLRP3-mediated pyroptosis by LPS transfection (Supplementary Figures S2A,B). Taken together, these results indicated that evodiamine pre-treatment specifically promoted NLRP3, but not NLRC4 or AIM2, inflammasome-mediated pyroptosis in macrophages.

### Evodiamine Induces α-Tubulin Acetylation and Mitochondrial Re-distribution

**FIGURE 3:**
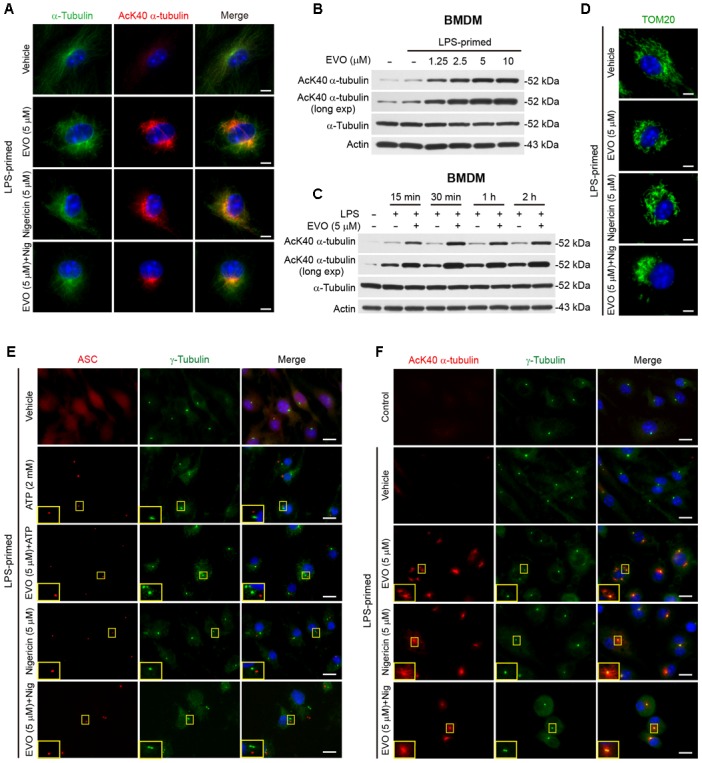
Evodiamine increased α-tubulin acetylation. BMDMs were primed with LPS (500 ng/ml) for 4 h, then treated with evodiamine (5 μM) for 1 h, followed by incubation with nigericin (5 μM) for 1 h **(A,D,E)** or ATP (2 mM) for 30 min **(F)** in the absence of LPS. **(A)** Representative immunofluorescence images showing α-tubulin (green) and acetylated (K40) α-tubulin (red) subcellular distributions. Nuclei (blue) were stained with Hoechst 33342. Scale bars, 2 μm. **(B,C)** LPS-primed BMDMs were treated with a grade does of evodiamine for 1 h **(B)** or evodiamine (5 μM) for indicated time periods **(C)** in the absence of LPS. Acetylated and total α-tubulin expression levels were determined by Western blotting. Actin was recruited as a loading control. **(D)** Representative images showing TOM20 (green) subcellular distribution. Scale bars, 2 μm. **(E)** Representative images showing acetylated α-tubulin (red) and γ-tubulin (green) subcellular distribution. The enlarged insets show the co-localization of acetylated α-tubulin and γ-tubulin in the perinuclear region. Scale bars, 10 μm. **(F)** Representative immunofluorescence images showing ASC (red) and γ-tubulin (green) subcellular distributions. The enlarged insets show the ASC and γ-tubulin in the perinuclear region. Scale bars, 10 μm. EVO, evodiamine.

In light of the cell morphological change (i.e., pseudopodia shrinkage) upon evodiamine treatment in LPS-primed cells, as observed above (Figure 2G), and previous findings that the microtubule transport machinery including α-tubulin acetylation has critical roles in regulating NLRP3 inflammasome activation (Misawa et al., 2013), we focused our investigation on whether evodiamine enhanced NLRP3 inflammasome activation by affecting the microtubule cytoskeleton. Immunofluorescence microscopy showed that evodiamine strikingly induced α-tubulin acetylation in LPS-primed macrophages, and the acetylated α-tubulin formed bundles in the perinuclear region of cells (Figure 3A). While nigericin stimulation also induced a bundle of acetylated α-tubulin in perinuclear region, evodiamine pre-treatment appeared to make this bundle more concentrated. Western blotting verified that evodiamine dose-dependently and rapidly induced α-tubulin acetylation in LPS-primed and unprimed macrophages (Figure 3B,C and Supplementary Figures S3A–C). Interestingly, evodiamine further increased the acetylation of α-tubulin in ATP- or nigericin-treated BMDMs (Supplementary Figures S4A,B). Considering the role of acetylated α-tubulin in cargo trafficking and NLRP3 inflammasome assembly [e.g., carrying ASC by mitochondria (Misawa et al., 2013)], we assessed whether evodiamine affected mitochondrial distribution. Immunofluorescence microscopy showed that the mitochondria distributed all over the cytosol in vehicle group, but upon evodiamine treatment, they were concentrated around the nucleus. Intriguingly, pre-treatment with evodiamine followed by nigericin treatment further promoted the aggregation of mitochondria, being located at one side of the perinuclear region (Figure 3D), suggesting that evodiamine had promoted the mitochondrial transport. Accompanying the re-distribution of mitochondria, evenly-distributed ASC (as observed in vehicle group) moved toward the microtubule organizing center (MTOCs, indicated by γ-tubulin) and concentrated into one or more specks upon ATP treatment, although most of the cells harbored only one ASC speck near the MTOC (Figure 3E). In line with previous studies showing the co-localization of NLRP3 specks with the MTOCs in PMA-differentiated THP-1 cells (Li et al., 2017c), evodiamine-induced bundles of acetylated α-tubulin were highly co-localized with the MTOCs (Figure 3F). Interestingly, several dots of γ-tubulin together with acetylated α-tubulin were observed in evodiamine-treated cells. Together, these results indicated that evodiamine induced α-tubulin acetylation, which might facilitate mitochondrion-mediated transport of ASC toward the minus end of the microtubules to enhance NLRP3 inflammasome assembly and activation.

### Blockade of α-Tubulin Acetylation Attenuates Evodiamine-Mediated Augmentation of NLRP3 Inflammasome Activation and Pyroptosis in Macrophages

**FIGURE 4:**
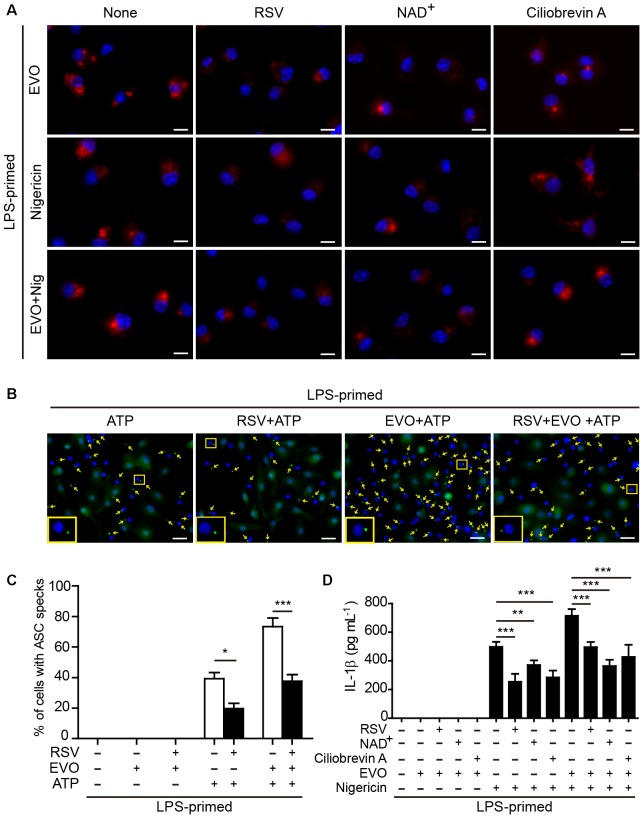
Evodiamine-mediated enhancement of ATP- or nigericin-induced NLRP3 inflammasome activation was attenuated by blocking acetylation of α-tubulin. **(A)** LPS-primed BMDMs were pre-treated with resveratrol (5 μM), NAD^+^ (10 μM) or ciliobrevin A (30 μM) for 30 min, and then incubated with evodiamine (5 μM) for 1 h, or followed by stimulation with nigericin (5 μM) for 1 h. Representative immunofluorescence images showing acetylated α-tubulin (red) subcellular distribution. Nuclei (blue) were revealed by Hoechst 33342. The images for acetylated α-tubulin and nuclei were captured, respectively, and merged together. Scale bars, 10 μm. **(B)** LPS-primed BMDMs were pre-treated with resveratrol (5 μM) for 30 min, and then incubated with indicated dose of evodiamine for 1 h, followed by stimulation with ATP (2 mM) for 30 min. Representative images showing ASC (green) subcellular distribution. The images for ASC and nuclei were captured, respectively, and merged together. Yellow arrows indicate ASC specks and the enlarged inset showing cells with an ASC speck. Scale bars, 20 μm. **(C)** Percentages of cells with an ASC speck relative to the total number of cells from 5 random fields (one field per well) each containing ∼200 cells. Data are shown as mean ± SD (*n* = 5). *^∗^P* < 0.05, ^∗∗∗^*P* < 0.001, two-tailed Student’s *t*-test. **(D)** BMDMs were treated as in Figure 4A. The levels of soluble IL-1β in culture supernatants were measured by cytometric bead array (CBA) assay. The experiments were performed three times independently. Data are shown as mean ± SD (*n* = 5). One-way ANOVA: *P* < 0.0001; Tukey *post hoc* test: ^∗∗^*P* < 0.01, ^∗∗∗^*P* < 0.001; RSV, resveratrol; EVO, evodiamine; Nig, nigericin.

To corroborate the role of acetylated α-tubulin in mediating evodiamine’s effects on NLRP3 activation, we used resveratrol and NAD^+^ (nicotinamide adenine dinucleotide) to block the acetylation of α-tubulin in macrophages. Consistent with published studies (Misawa et al., 2013; Misawa et al., 2015), both resveratrol and NAD^+^ supplementations were able to suppress α-tubulin acetylation induced by either evodiamine, nigericin or their combination (Figure 4A). In agreement with the attenuation of acetylated α-tubulin, evodiamine-mediated enhancement of ASC speck formation upon ATP or nigericin stimulation was abrogated by resveratrol (Figure 4B,C and Supplementary Figures S5A,B). ATP-induced formation of ASC speck was also attenuated by resveratrol, in agreement with published studies (Misawa et al., 2013). Bead-based assay showed that evodiamine-induced increase in IL-1β release from the cells stimulated with ATP or nigericin was also abrogated by either resveratrol or NAD^+^ (Figure 4D). Since the activity of α-tubulin deacetylase SIRT2 is dependent on NAD^+^, we detected whether evodiamine decreased the intracellular NAD^+^ levels by using the NAD^+^/NADH assay kit. The results showed that evodiamine, either in the presence or absence of LPS, did not significantly change NAD^+^ levels (Supplementary Figure S6). As the trafficking of ASC on mitochondria along the microtubule track toward the minus end was mediated by the motor protein dynein (Kardon and Vale, 2009; Misawa et al., 2013), we next investigated the involvement of dynein in evodiamine-enhanced activation of the NLRP3 inflammasome by using ciliobrevin-A, a specific inhibitor of dynein (Firestone et al., 2012). As shown in Figure 4D, ciliobrevin-A pre-treatment not only suppressed nigericin-induced release of IL-1β but also abrogated evodiamine’s effect on enhancing IL-1β release from the macrophages upon nigericin stimulation, although it did not alter α-tubulin acetylation (Figure 4A). Taken together, these results indicated that evodiamine-induced augmentation of NLRP3 inflammasome activation was mediated by induction of α-tubulin acetylation thus facilitating dynein-mediated cargo transport of NLRP3 inflammasome components along the microtubule track.

### Knockdown of α-Tubulin *N*-Acetyltransferase αTAT1 Attenuates Evodiamine-Mediated Augmentation of NLRP3 Inflammasome Activation and Pyroptosis

**FIGURE 5:**
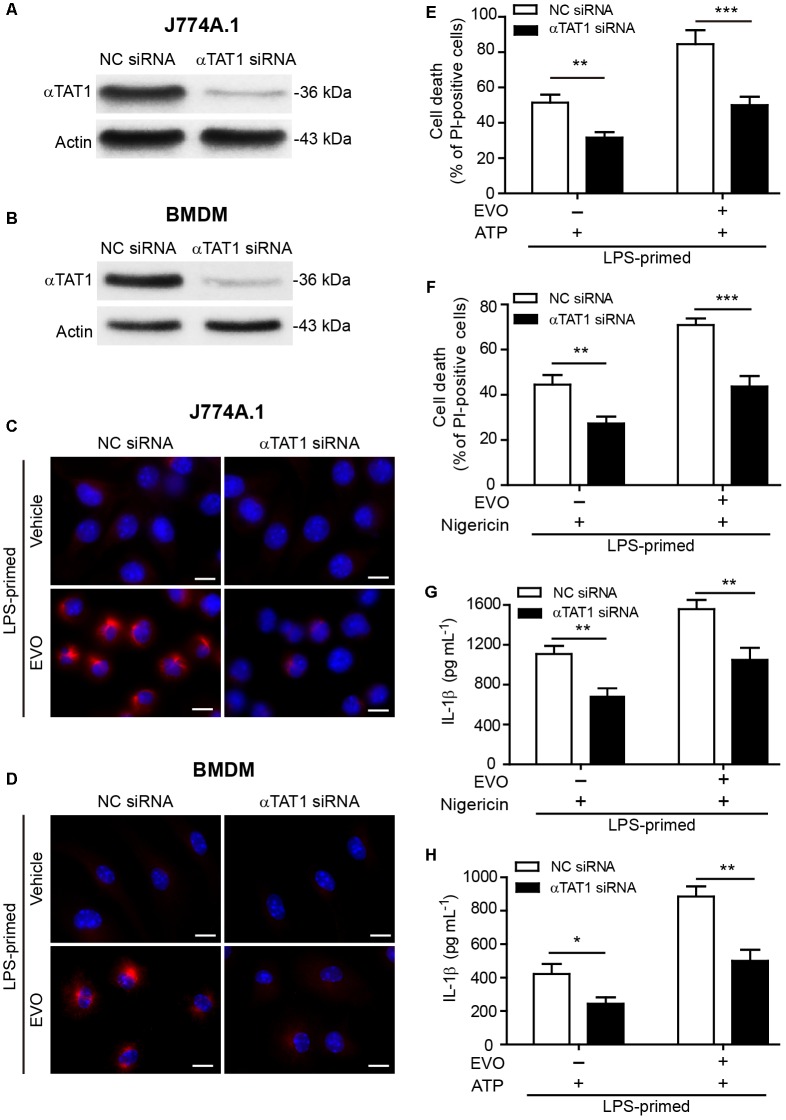
Knockdown of αTAT1 attenuated evodiamine-mediated enhancement of ATP- or nigericin-induced NLRP3 inflammasome activation and pyroptosis in macrophages. **(A–D)** J774A.1 cells and BMDMs were knocked down by small-interfering RNA (siRNA) targeting *αTAT1* gene (αTAT1 siRNA). Negative control siRNA (NC siRNA) was recruited as a knockdown control. 72 h after knockdown, J774A.1 cells **(A)** and BMDMs **(B)** were lysed and analyzed by Western blotting. Actin was used as a loading control for cell lysates. After *αTAT1* knockdown, J774A.1 cells **(C)** and BMDMs **(D)** were primed with LPS (500 ng/ml) for 4 h, then treated with evodiamine (5 μM) for 1 h. Representative immunofluorescence images showing acetylated α-tubulin (red) subcellular distribution. Nuclei (blue) were revealed by Hoechst 33342. The images for acetylated α-tubulin and nuclei were captured, respectively, and merged together. Scale bars, 10 μm. **(E–H)** J774A.1 cells **(E,G)** and BMDMs **(F,H)** were treated with *αTAT1* siRNA as in a. Cells were primed with LPS (500 ng/ml) for 4 h and then pre-treated with evodiamine (5 μM) for 1 h, followed by incubation with ATP (3 mM) for 1 h **(E)** or nigericin (5 μM) for 1 h **(F)** in the absence of LPS. **(E,F)** Cell death was measured by staining with propidium iodide (PI) (red, staining dead cells) and Hoechst 33342 (blue, staining total cells) together for 10 min. PI-positive cells were quantified by counting 5 randomly chosen fields (one field per well) containing around 100 cells each. **(G,H)** The levels of soluble IL-1β in the culture supernatants were detected by cytometric bead array assay. The experiments were performed three times independently. Data are shown as mean ± SD (*n* = 5). *^∗^P* < 0.05, ^∗∗^*P* < 0.01, ^∗∗∗^*P* < 0.001, by two-tailed Student’s *t*-test. EVO, evodiamine.

To further confirm the role of acetylated α-tubulin in mediating evodiamine’s effect on enhancing NLRP3 activation, siRNA was recruited to knock down the expression of αTAT1 (MEC-17), the acetyltransferase responsible for α-tubulin acetylation (Akella et al., 2010; Kalebic et al., 2013; Misawa et al., 2013). Western blotting and immunofluorescence microscopy showed that αTAT1 expression was markedly decreased by *αTAT1*-specific siRNA compared to the negative control (Figure 5A,B and Supplementary Figure S7A). Knockdown of *αTAT1* abrogated evodiamine-induced increase in α-tubulin acetylation in both J774A.1 cells and BMDMs (Figure 5C,D). Meanwhile, in the absence of evodiamine stimulation, the basal acetylation level of α-tubulin was also decreased after *αTAT1* knockdown (Supplementary Figures S7B,C). In agreement with the abrogation of α-tubulin acetylation, *αTAT1* knockdown significantly attenuated the effects of evodiamine in augmentation of ATP- or nigericin-induced pyroptosis (Figure 5E,F) and IL-1β release (Figure 5G,H). Altogether, these results indicated that αTAT1 and α-tubulin acetylation had been involved in evodiamine-mediated augmentation of NLRP3 inflammasome activation and pyroptosis by ATP or nigericin treatment in macrophages.

### Evodiamine Enhances NLRP3Inflammasome Activation *in vivo* andProtects Mice Against Bacterial Infection

**FIGURE 6:**
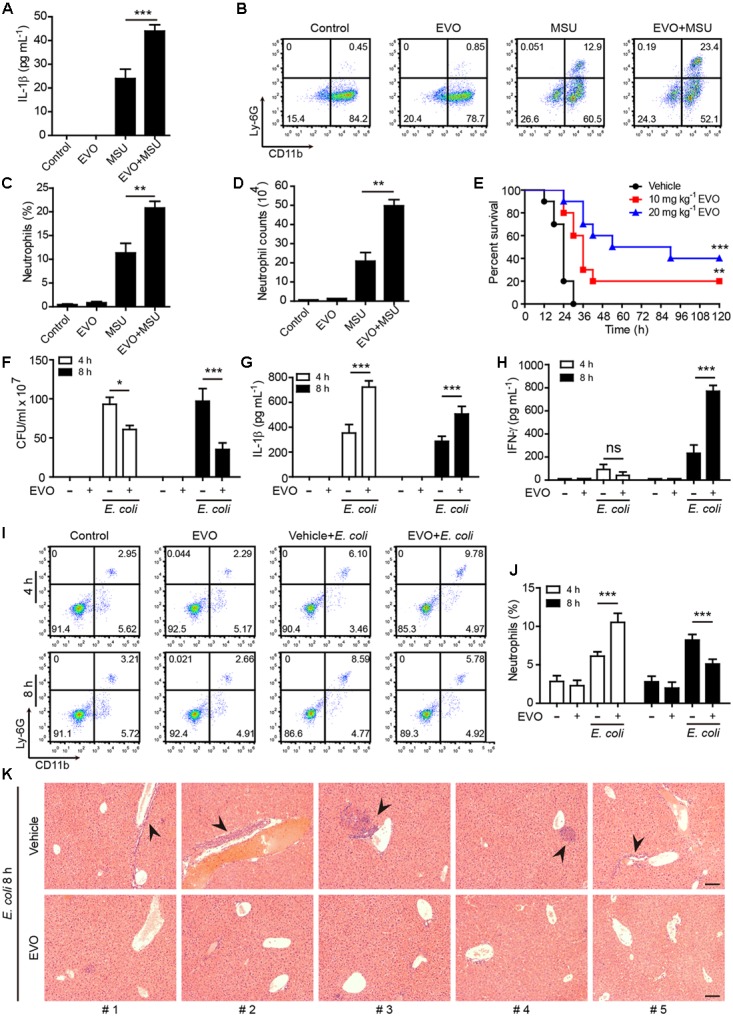
Evodiamine enhanced NLRP3 inflammasome activation *in vivo* and protected mice against bacterial infection. **(A)** C57BL/6 mice were administered intragastrically (i.g.) with evodiamine (20 mg/kg body weight) or vehicle (2% Tween-80 in PBS) for 1 h before intraperitoneally injected with MSU (1 mg per mouse). After 6 h, peritoneal levels of IL-1β were measured by cytometric bead array assay (five mice per group). **(B–D)** Neutrophils (CD11b^+^Ly-6G^+^) in the peritoneal cavity after MSU challenge were analyzed by flow cytometry. Representative set of flow cytometric dot-plots are shown in **(B)**. **(C)** Quantification of CD11b^+^Ly-6G^+^ cells in **(B)**. **(D)** The numbers of neutrophils were calculated by their percentages times the total peritoneal cell numbers (determined by a hemocytometer), respectively. Data are expressed as mean ± SD (*n* = 5). One-way analysis of variance (ANOVA): *P* < 0.0001 **(A,C,D)**; Tukey *post hoc* test: ^∗∗^*P* < 0.01, ^∗∗∗^*P* < 0.001. **(E)** Mice were administered (i.g.) with evodiamine (10 or 20 mg/kg body weight) or vehicle (2% Tween-80 in PBS) once 3 h before intraperitoneal injection with viable *E. coli* (2 × 10^9^ CFU/mouse). One hour after the bacterial infection, mice were intragastrically administered with evodiamine or vehicle once again. Mouse survival was monitored every 6 h for five consecutive days. Kaplan–Meier survival curves were used to analyze the data (10 mice per group). The significance was evaluated by the log-rank test. ^∗∗^*P* < 0.01, ^∗∗∗^*P* < 0.001, versus vehicle. **(F)** Bacterial counts in the peritoneal cavity at 4 and 8 h post bacterial infection were measured by using an ultraviolet-visible spectrophotometer, and the corresponding colony-forming units (CFUs) were determined on lysogeny broth (LB) media agar plates. **(G,H)** The serum levels of IL-1β and IFN-γ at 4 and 8 h post bacterial infection was measured by cytometric bead array assay (five mice per group). **(I,J)** Neutrophils (CD11b^+^Ly-6G^+^) in the peripheral blood after bacterial infection were analyzed by flow cytometry as did in **(B,C)** (*n* = 5 mice). One-way ANOVA: *P* < 0.0001 **(F–J)**; Tukey *post hoc* test: ^∗∗∗^*P* < 0.001. EVO, evodiamine. **(K)** Hematoxylin and eosin staining of the liver sections of mice infected with live *E. coli* for 8 h. Representative images from each mouse were presented and arrowheads indicated infiltrated inflammatory cells. The numbers at the bottom indicate mouse number. Scale bars, 100 μm.

We next assessed whether evodiamine could enhance NLRP3-dependent inflammation *in vivo*. Intraperitoneal injection of MSU crystals elicits NLRP3-dependent inflammatory responses in mouse peritoneal cavity, characterized by IL-1β production and massive neutrophil influx (Martinon et al., 2006; Misawa et al., 2015). Consistent with the results observed in macrophages *in vitro*, evodiamine administration significantly enhanced MSU-induced IL-1β production (Figure 6A) and neutrophil recruitment *in vivo* (Figure 6B–D), indicating that evodiamine was able to promote NLRP3 inflammasome activation in mice *in vivo*.

As published studies have shown that enhanced caspase-1 activation and IL-1β production play critical roles in antibacterial defenses (Joshi et al., 2002; Saleh et al., 2006; Yang F.M. et al., 2018), we next explored the functional relevance of evodiamine in enhancing the innate immunity in a mouse model of bacterial infection. Mice were administered intragastrically with evodiamine or vehicle followed by intraperitoneal injection with a lethal dose of viable *E. coli* (2 × 10^9^ CFU/mouse). Evodiamine or vehicle was given once again after the bacterial infection. Survival analysis showed that all vehicle-treated mice succumbed to such a lethal dose of *E. coli* infection within 30 h, whereas 20% and 40% of evodiamine-administered mice survived the period of observation (120 h), respectively (Figure 6E). In a paralleled experiment, evodiamine significantly decreased the bacterial load (CFUs) in the peritoneal cavity when compared with vehicle (Figure 6F), indicating enhanced clearance of bacteria by evodiamine treatment. The serum IL-1β levels were markedly increased both at 4 h and 8 h post infection as compared with vehicle (Figure 6G), suggesting that evodiamine had promoted NLRP3 activation in the mouse model. Notably, evodiamine administration also markedly increased serum production of IFN-γ and TNF-α at 8 h post infection although their levels were unchanged (IFN-γ) or slightly decreased (TNF-α) at 4 h post infection as compared with vehicle treatment (Figure 6H and Supplementary Figure S8A). Besides, evodiamine administration had weakly affected serum IL-10, IL-6, CCL2 (MCP-1), and IL-12p70 (Supplementary Figures S8B–E). Accompanying increased production of IL-1β, the percentages of neutrophils (CD11b^+^ Ly-6G^+^) in the blood of evodiamine group were significantly increased at 4 h post infection as compared with those in vehicle group (Figure 6I,J). However, the percentages of neutrophils were markedly reduced by evodiamine at 8 h post infection (Figure 6I,J), which was likely due to clearance of bacteria (Figure 6F) thus dampening the inflammatory response. In addition, histopathological analysis revealed that bacterial infection resulted in overt infiltration of inflammatory cells in the liver in vehicle group whereas evodiamine-administered mice did not have apparent infiltration of inflammatory cells (Figure 6K), suggesting that evodiamine had improved systemic injury caused by bacterial infection. Together, these results suggested that evodiamine conferred antibacterial defenses by promoting NLRP3-mediated production of IL-1β and other inflammatory cytokines (such as IFN-γ and TNF-α).

### Resveratrol Treatment Abrogates Evodiamine-Mediated Augmentation of Antibacterial Defenses in Mice *in vivo*

**FIGURE 7:**
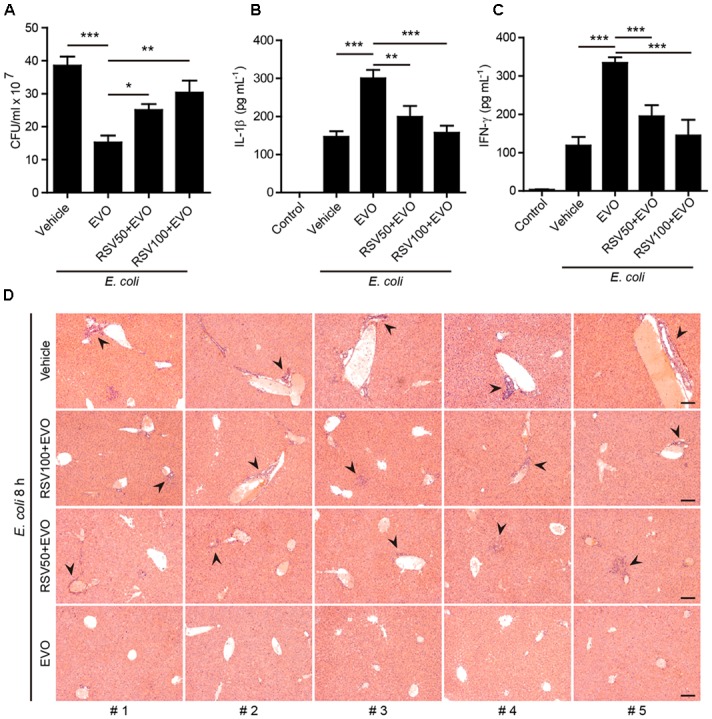
Resveratrol treatment reversed evodiamine-mediated antibacterial effects *in vivo*. Mice were administered intragastrically (i.g.) with resveratrol solution (50 or 100 mg/kg body weight) or vehicle (2% Tween-80 in PBS). After 30 min, mice were administered (i.g.) with evodiamine (20 mg/kg body weight) or vehicle (2% Tween-80 in PBS) once 3 h before intraperitoneal injection with viable *E. coli* (1 × 10^9^ CFU/mouse). One hour after the bacterial infection, mice were administered (i.g.) with evodiamine or vehicle once again. **(A)** Bacterial counts in the peritoneal cavity at 8 h post bacterial infection was measured by using an ultraviolet-visible spectrophotometer, and the corresponding colony-forming units (CFUs) were determined on lysogeny broth (LB) media agar plates. **(B,C)** The serum levels of IL-1β and IFN-γ at 8 h post bacterial infection was measured by cytometric bead array assay (five mice per group). Data are expressed as mean ± SD (*n* = 5). One-way ANOVA: *P* < 0.0001 **(A–C)**; Tukey *post hoc* test: ^∗^*P* < 0.05, ^∗∗^*P* < 0.01, ^∗∗∗^*P* < 0.001. EVO, evodiamine; RSV, resveratrol. **(D)** Hematoxylin and eosin staining of the livers sections of mice infected with live *E. coli* for 8 h. Representative images from each mouse were presented and arrowheads indicated infiltrated inflammatory cells. The numbers at the bottom indicate mouse number. Scale bars, 100 μm.

As resveratrol treatment counteracted the effect of evodiamine in inducing α-tubulin acetylation and augmenting NLRP3 inflammasome activation *in vitro*, we finally explored whether resveratrol could reverse evodiamine-mediated augmentation of innate immune responses against bacterial infection *in vivo*. To this end, mice were gavaged with resveratrol solution or vehicle followed by evodiamine administration and bacterial infection. The results showed that evodiamine-mediated reduction of bacterial burden in the peritoneal cavity was attenuated by resveratrol pre-treatment (Figure 7A). Notably, mice pre-treated with resveratrol reversed evodiamine-enhanced production of serum IL-1β, IFN-γ (Figure 7B–C), TNF-α and IL-10 (Supplementary Figures S9A,B). However, treatment with resveratrol did not change the levels CCL2 and IL-12p70 or slightly changed the levels of IL-6, upon bacterial infection (Supplementary Figures S9C–E). Histopathological analysis also demonstrated that resveratrol weakened the protective effect of evodiamine in attenuating the infiltration of inflammatory cells in the liver of bacterial infected mice. Together, these results indicated that evodiamine-mediated augmentation of innate immune responses and antibacterial defenses was abrogated by resveratrol *in vivo*.

## Discussion

The microtubule cytoskeleton has critical roles in regulating NLRP3 inflammasome activation by controlling the spatial rearrangement and transport of NLRP3 inflammasome components (Misawa et al., 2013; Li et al., 2017c). Accordingly, microtubule-destabilizing agents, such as colchicine (Gigant et al., 2009), suppress the activation of NLRP3 inflammasome both *in vitro* and *in vivo* (Martinon et al., 2006; Dalbeth et al., 2014), which may explain the underlying mechanism for colchicine’s application in the treatment of NLRP3 activation related diseases such as gout and familial Mediterranean fever (Kiraz et al., 1998; Martinon et al., 2006). However, it is unknown whether evodiamine, an agent stabilizing microtubules (Huang et al., 2004; Liao et al., 2005), influences NLRP3 inflammasome. In this study, we found that evodiamine augmented the activation of NLRP3 inflammasome in macrophages induced by extracellular ATP, nigericin or intracellularly transfected LPS. It also enhanced MSU-induced NLRP3 activation *in vivo*. Moreover, evodiamine administration conferred intensified innate defense against bacterial infection, thereby increasing the survival rate of mice infected with a lethal dose of *E. coli*. Thus, in contrast to microtubule-destabilizing agents like colchicine, the phytochemical evodiamine, likely acting as microtubule-stabilizing agent in macrophages, promoted NLRP3 inflammasome activation leading to augmented antibacterial innate immune responses.

One major concern of this study is the underlying mechanism(s) for evodiamine-mediated enhancement of NLRP3 inflammasome activation. It has been shown that acetylation of α-tubulin in the microtubule cytoskeleton facilitates transport of NLRP3 inflammasome components (e.g., ASC) on mitochondria to the perinuclear region of the cell to promote the inflammasome assembly and activation (Misawa et al., 2013; Li et al., 2017c). Considering that evodiamine can bind to and stabilize the microtubule cytoskeleton (Liao et al., 2005), we hypothesized that microtubules might have been involved in evodiamine-mediated enhancement of NLRP3 inflammasome activation. In support of this notion, we found that evodiamine induced the rearrangement of microtubules, consistent with previous study (Liao et al., 2005). Interestingly, evodiamine treatment alone markedly increased the acetylation of α-tubulin in microtubules to form two or more bundles co-localized with the MTOCs (indicated by γ-tubulin) within the cell, and evodiamine combined with NLRP3 inflammasome inducer further increased α-tubulin acetylation to form one acetylated α-tubulin bundle co-localized with the MTOC, concurrent with enhanced aggregation of mitochondria in the perinuclear region during the NLRP3 activation. Induction of multiple MTOCs together with highly acetylated microtubules by evodiamine might have accelerated the cargo (e.g., mitochondrion) trafficking of NLRP3 inflammasome components, carrying the evenly-distributed ASC from several routes to move toward the MTOCs and concentrate into specks upon NLRP3 inflammasome stimuli, even though finally only one ASC speck could be observed. It seemed that the MTOC-oriented trafficking of ASC thus forming into a speck is necessary for NLRP3 inflammasome activation. Supporting this, previous studies have demonstrated that the proper positioning of ASC and NLRP3 can be regulated by microtubule-affinity regulating kinase 4 (MARK4), and the apposition of NLRP3, MARK4 and γ-tubulin has been observed upon NLRP3 inflammasome activation (Li et al., 2017c). Therefore, evodiamine is likely to augment NLRP3 inflammasome activation through inducing α-tubulin acetylation.

To further support the role of α-tubulin acetylation in mediating enhanced NLRP3 activation by evodiamine, we used pharmacological agents or genetic knockdown to block the α-tubulin acetylation. Published studies have shown that NAD^+^ or resveratrol can efficiently suppress α-tubulin acetylation in macrophages during NLRP3 activation (Misawa et al., 2013, 2015). Consistently, we showed that NAD^+^ or resveratrol not only significantly attenuated ATP- or nigericin-induced NLRP3 activation but also diminished evodiamine-mediated enhancement of NLRP3 activation, concomitant with the markedly-decreased acetylation of α-tubulin in microtubules. However, our experiments showed that evodiamine did not change the intracellular NAD^+^ levels, suggesting that, although NAD^+^-dependent α-tubulin deacetylase takes part in the balance of α-tubulin acetylation, it might not be regulated by evodiamine via affecting intracellular NAD^+^ levels. Besides, dynein-specific inhibitor ciliobrevin A (Firestone et al., 2012) abrogated nigericin-induced release of IL-1β, either in the presence or absence of evodiamine. Moreover, knockdown of αTAT1 expression also diminished evodiamine-mediated augmentation of NLRP3 activation and pyroptosis, concurrent with reduced α-tubulin acetylation, suggesting the involvement of αTAT1 in these events. All these results indicated that evodiamine augmented NLRP3 inflammasome activation by increasing the acetylation of α-tubulin thereby facilitating transport of NLRP3 inflammasome components along the microtubule tracks.

Inflammasome activation is a critical defense mechanism of the innate immune system against microbial infection (Lamkanfi and Dixit, 2014). One of the outcomes of the inflammasome activation is the maturation and release of IL-1β, as well as other inflammatory cytokines. These cytokines not only promote the functions of phagocytes by increasing their phagocytic and bacterial killing abilities (Piccini et al., 2008), but also are necessary for the recruitment of other immune cells including neutrophils and monocytes/macrophages (Bauman et al., 2009; Guarda et al., 2011). Consequently, induction of inflammasome activation and IL-1β release can intensify the host defense against bacterial infection in murine models (Guarda et al., 2011; Descamps et al., 2012; Wegiel et al., 2014). Consistent with enhanced activation of NLRP3 inflammasome *in vitro*, evodiamine treatment efficiently enhanced MSU-induced IL-1β production and neutrophil influx *in vivo* in mice. In the mouse model of bacterial infection, evodiamine efficiently increased the serum levels of IL-1β, TNF-α and IFN-γ, thus potentiating the innate immune responses to enhance the clearance of bacteria and dampening the infiltration of inflammatory cells in the liver. Consistent with these observations, evodiamine administration significantly improved the survival of mice infected with a lethal dose of live *E. coli*. Interestingly, resveratrol administration antagonized the action of evodiamine in increasing IL-1β, TNF-α and IFN-γ production, accompanied by decreased bacterial clearance in mice. Our findings are in agreement with published studies showing that unleashing innate immune signaling confers enhanced bacterial clearance and bacterial sepsis resistance by increased secretion of IL-1β and IFN-γ in serum (Saleh et al., 2006; Yang F.M. et al., 2018). Other studies have shown that the IFN-γ pathway has a crucial role in mediating the survival advantage of septic caspase-12-deficient mice with unleashed innate responses (Guarda et al., 2011). IFN-γ is an established survival factor in sepsis of both humans and mice (Zantl et al., 1998; Hotchkiss et al., 2003; Prass et al., 2003). Although additional investigation is needed to clarify the role INF-γ in the current experimental setting, our findings indicated that evodiamine endowed an intensified innate response against bacterial infection likely by enhancing NLRP3 inflammasome activation in mice.

In summary, we found in this study that the phytochemical evodiamine was able to target the microtubule cytoskeleton by inducing acetylation at K40 residue of α-tubulin, thereby facilitating the transportation of inflammasome components along the microtubules and potentiating the activation of NLRP3 inflammasome activation both *in vitro* and *in vivo* in mice. We also found that evodiamine administration was able to confer enhanced innate responses in mice against bacterial infection. Further investigations are needed to elucidate the precise mechanism for evodiamine-mediated action on microtubules. Nevertheless, our findings highlight the potential application of evodiamine, by induction of microtubule acetylation, in enhancing antimicrobial responses in patients with pathogen infection.

## Author Contributions

C-GL and Q-ZZ performed the *in vitro* studies. M-YC, L-HX, C-CZ, C-YZ, and F-YM conducted the animal studies. C-GL analyzed the data. D-YO and X-HH supervised the study and wrote the manuscript.

## Conflict of Interest Statement

The authors declare that the research was conducted in the absence of any commercial or financial relationships that could be construed as a potential conflict of interest.

## References

[B1] Akella J. S. Wloga D. Kim J. Starostina N. G. Lyons-Abbott S. Morrissette N. S. (2010). MEC-17 is an alpha-tubulin acetyltransferase. *Nature* 467 218–222. 10.1038/nature09324 20829795 PMC2938957

[B2] Balabanian L. Berger C. L. Hendricks A. G. (2017). Acetylated microtubules are preferentially bundled leading to enhanced kinesin-1 motility. *Biophys. J.* 113 1551–1560. 10.1016/j.bpj.2017.08.009 28978447 PMC5627185

[B3] Bauman D. R. Bitmansour A. D. Mcdonald J. G. Thompson B. M. Liang G. Russell D. W. (2009). 25-Hydroxycholesterol secreted by macrophages in response to Toll-like receptor activation suppresses immunoglobulin A production. *Proc. Natl. Acad. Sci. U.S.A.* 106 16764–16769. 10.1073/pnas.0909142106 19805370 PMC2757821

[B4] Broz P. Dixit V. M. (2016). Inflammasomes: mechanism of assembly, regulation and signalling. *Nat. Rev. Immunol.* 16 407–420. 10.1038/nri.2016.58 27291964

[B5] Bryant C. Fitzgerald K. A. (2009). Molecular mechanisms involved in inflammasome activation. *Trends Cell Biol.* 19 455–464. 10.1016/j.tcb.2009.06.002 19716304

[B6] Chung S. W. Liu X. Macias A. A. Baron R. M. Perrella M. A. (2008). Heme oxygenase-1-derived carbon monoxide enhances the host defense response to microbial sepsis in mice. *J. Clin. Invest.* 118 239–247. 10.1172/JCI32730 18060048 PMC2104480

[B7] Coll R. C. Robertson A. A. Chae J. J. Higgins S. C. Munoz-Planillo R. Inserra M. C. (2015). A small-molecule inhibitor of the NLRP3 inflammasome for the treatment of inflammatory diseases. *Nat. Med.* 21 248–255. 10.1038/nm.3806 25686105 PMC4392179

[B8] Dalbeth N. Lauterio T. J. Wolfe H. R. (2014). Mechanism of action of colchicine in the treatment of gout. *Clin. Ther.* 36 1465–1479. 10.1016/j.clinthera.2014.07.017 25151572

[B9] Descamps D. Le Gars M. Balloy V. Barbier D. Maschalidi S. Tohme M. (2012). Toll-like receptor 5 (TLR5), IL-1beta secretion, and asparagine endopeptidase are critical factors for alveolar macrophage phagocytosis and bacterial killing. *Proc. Natl. Acad. Sci. U.S.A.* 109 1619–1624. 10.1073/pnas.1108464109 22307620 PMC3277124

[B10] Firestone A. J. Weinger J. S. Maldonado M. Barlan K. Langston L. D. O’donnell M. (2012). Small-molecule inhibitors of the AAA+ ATPase motor cytoplasmic dynein. *Nature* 484 125–129. 10.1038/nature10936 22425997 PMC3321072

[B11] Ge X. Chen S. Liu M. Liang T. Liu C. (2015). Evodiamine attenuates PDGF-BB-induced migration of rat vascular smooth muscle cells through activating PPARgamma. *Int. J. Mol. Sci.* 16 28180–28193. 10.3390/ijms161226093 26703570 PMC4691040

[B12] Gigant B. Cormier A. Dorleans A. Ravelli R. B. Knossow M. (2009). Microtubule-destabilizing agents: structural and mechanistic insights from the interaction of colchicine and vinblastine with tubulin. *Top. Curr. Chem.* 286 259–278. 10.1007/128_2008_11 23563615

[B13] Guarda G. Braun M. Staehli F. Tardivel A. Mattmann C. Forster I. (2011). Type I interferon inhibits interleukin-1 production and inflammasome activation. *Immunity* 34 213–223. 10.1016/j.immuni.2011.02.006 21349431

[B14] Guo C. Xie S. Chi Z. Zhang J. Liu Y. Zhang L. (2016). Bile acids control inflammation and metabolic disorder through inhibition of NLRP3 inflammasome. *Immunity* 45 802–816. 10.1016/j.immuni.2016.09.008 27692610

[B15] Guo W. Liu W. Chen Z. Gu Y. Peng S. Shen L. (2017). Tyrosine phosphatase SHP2 negatively regulates NLRP3 inflammasome activation via ANT1-dependent mitochondrial homeostasis. *Nat. Commun.* 8:2168. 10.1038/s41467-017-02351-0 29255148 PMC5735095

[B16] He W. T. Wan H. Hu L. Chen P. Wang X. Huang Z. (2015). Gasdermin D is an executor of pyroptosis and required for interleukin-1beta secretion. *Cell Res.* 25 1285–1298. 10.1038/cr.2015.139 26611636 PMC4670995

[B17] He Y. Hara H. Nunez G. (2016). Mechanism and regulation of NLRP3 inflammasome activation. *Trends Biochem. Sci.* 41 1012–1021. 10.1016/j.tibs.2016.09.002 27669650 PMC5123939

[B18] He Y. Varadarajan S. Munoz-Planillo R. Burberry A. Nakamura Y. Nunez G. (2014). 3,4-methylenedioxy-beta-nitrostyrene inhibits NLRP3 inflammasome activation by blocking assembly of the inflammasome. *J. Biol. Chem.* 289 1142–1150. 10.1074/jbc.M113.515080 24265316 PMC3887181

[B19] Hotchkiss R. S. Chang K. C. Grayson M. H. Tinsley K. W. Dunne B. S. Davis C. G. (2003). Adoptive transfer of apoptotic splenocytes worsens survival, whereas adoptive transfer of necrotic splenocytes improves survival in sepsis. *Proc. Natl. Acad. Sci. U.S.A.* 100 6724–6729. 10.1073/pnas.1031788100 12736377 PMC164514

[B20] Huang D. M. Guh J. H. Huang Y. T. Chueh S. C. Chiang P. C. Teng C. M. (2005). Induction of mitotic arrest and apoptosis in human prostate cancer pc-3 cells by evodiamine. *J. Urol.* 173 256–261. 10.1097/01.ju.0000141587.72429.e3 15592092

[B21] Huang Y. C. Guh J. H. Teng C. M. (2004). Induction of mitotic arrest and apoptosis by evodiamine in human leukemic T-lymphocytes. *Life Sci.* 75 35–49. 10.1016/j.lfs.2003.11.025 15102520

[B22] Hubbert C. Guardiola A. Shao R. Kawaguchi Y. Ito A. Nixon A. (2002). HDAC6 is a microtubule-associated deacetylase. *Nature* 417 455–458. 10.1038/417455a 12024216

[B23] Iwasaki A. Medzhitov R. (2015). Control of adaptive immunity by the innate immune system. *Nat. Immunol.* 16 343–353. 10.1038/ni.3123 25789684 PMC4507498

[B24] Jiang X. H. Wu Q. Q. Xiao Y. Yuan Y. Yang Z. Bian Z. Y. (2017). Evodiamine prevents isoproterenol-induced cardiac fibrosis by regulating endothelial-to-mesenchymal transition. *Planta Med.* 83 761–769. 10.1055/s-0042-124044 28010025

[B25] Jo E. K. Kim J. K. Shin D. M. Sasakawa C. (2016). Molecular mechanisms regulating NLRP3 inflammasome activation. *Cell Mol. Immunol.* 13 148–159. 10.1038/cmi.2015.95 26549800 PMC4786634

[B26] Joshi V. D. Kalvakolanu D. V. Hebel J. R. Hasday J. D. Cross A. S. (2002). Role of caspase 1 in murine antibacterial host defenses and lethal endotoxemia. *Infect. Immun.* 70 6896–6903. 10.1128/IAI.70.12.6896-6903.2002 12438367 PMC133093

[B27] Kalebic N. Sorrentino S. Perlas E. Bolasco G. Martinez C. Heppenstall P. A. (2013). alphaTAT1 is the major alpha-tubulin acetyltransferase in mice. *Nat. Commun.* 4:1962. 10.1038/ncomms2962 23748901

[B28] Kardon J. R. Vale R. D. (2009). Regulators of the cytoplasmic dynein motor. *Nat. Rev. Mol. Cell Biol.* 10 854–865. 10.1038/nrm2804 19935668 PMC3394690

[B29] Kayagaki N. Warming S. Lamkanfi M. Vande Walle L. Louie S. Dong J. (2011). Non-canonical inflammasome activation targets caspase-11. *Nature* 479 117–121. 10.1038/nature10558 22002608

[B30] Kayagaki N. Wong M. T. Stowe I. B. Ramani S. R. Gonzalez L. C. Akashi-Takamura S. (2013). Noncanonical inflammasome activation by intracellular LPS independent of TLR4. *Science* 341 1246–1249. 10.1126/science.1240248 23887873

[B31] Kiraz S. Ertenli I. Arici M. Calguneri M. Haznedaroglu I. Celik I. (1998). Effects of colchicine on inflammatory cytokines and selectins in familial Mediterranean fever. *Clin. Exp. Rheumatol.* 16 721–724. 9844766

[B32] Lamkanfi M. Dixit V. M. (2014). Mechanisms and functions of inflammasomes. *Cell* 157 1013–1022. 10.1016/j.cell.2014.04.007 24855941

[B33] Li C. G. Yan L. Jing Y. Y. Xu L. H. Liang Y. D. Wei H. X. (2017a). Berberine augments ATP-induced inflammasome activation in macrophages by enhancing AMPK signaling. *Oncotarget* 8 95–109. 10.18632/oncotarget.13921 27980220 PMC5352208

[B34] Li C. G. Yan L. Mai F. Y. Shi Z. J. Xu L. H. Jing Y. Y. (2017b). Baicalin Inhibits NOD-Like receptor family, pyrin containing domain 3 inflammasome activation in murine macrophages by augmenting protein kinase a signaling. *Front. Immunol.* 8:1409. 10.3389/fimmu.2017.01409 29163487 PMC5674921

[B35] Li X. Thome S. Ma X. Amrute-Nayak M. Finigan A. Kitt L. (2017c). MARK4 regulates NLRP3 positioning and inflammasome activation through a microtubule-dependent mechanism. *Nat. Commun.* 8:15986. 10.1038/ncomms15986 28656979 PMC5493753

[B36] Liao C. H. Pan S. L. Guh J. H. Chang Y. L. Pai H. C. Lin C. H. (2005). Antitumor mechanism of evodiamine, a constituent from Chinese herb Evodiae fructus, in human multiple-drug resistant breast cancer NCI/ADR-RES cells in vitro and in vivo. *Carcinogenesis* 26 968–975. 10.1093/carcin/bgi041 15705600

[B37] Liao J. F. Chiou W. F. Shen Y. C. Wang G. J. Chen C. F. (2011). Anti-inflammatory and anti-infectious effects of Evodia rutaecarpa (Wuzhuyu) and its major bioactive components. *Chin. Med.* 6:6. 10.1186/1749-8546-6-6 21320305 PMC3046897

[B38] Liu Y. Jing Y. Y. Zeng C. Y. Li C. G. Xu L. H. Yan L. (2017). Scutellarin suppresses NLRP3 inflammasome activation in macrophages and protects mice against bacterial sepsis. *Front. Pharmacol.* 8:975. 10.3389/fphar.2017.00975 29375379 PMC5767189

[B39] Liu Y. N. Pan S. L. Liao C. H. Huang D. Y. Guh J. H. Peng C. Y. (2009). Evodiamine represses hypoxia-induced inflammatory proteins expression and hypoxia-inducible factor 1alpha accumulation in RAW264.7. *Shock* 32 263–269. 10.1097/SHK.0b013e31819940cb 19106818

[B40] Man S. M. Kanneganti T. D. (2015). Regulation of inflammasome activation. *Immunol. Rev.* 265 6–21. 10.1111/imr.12296 25879280 PMC4400844

[B41] Martinon F. Petrilli V. Mayor A. Tardivel A. Tschopp J. (2006). Gout-associated uric acid crystals activate the NALP3 inflammasome. *Nature* 440 237–241. 10.1038/nature04516 16407889

[B42] Meng Z. J. Wu N. Liu Y. Shu K. J. Zou X. Zhang R. X. (2015). Evodiamine inhibits the proliferation of human osteosarcoma cells by blocking PI3K/Akt signaling. *Oncol. Rep.* 34 1388–1396. 10.3892/or.2015.4084 26135006

[B43] Misawa T. Saitoh T. Kozaki T. Park S. Takahama M. Akira S. (2015). Resveratrol inhibits the acetylated alpha-tubulin-mediated assembly of the NLRP3-inflammasome. *Int. Immunol.* 27 425–434. 10.1093/intimm/dxv018 25855661

[B44] Misawa T. Takahama M. Kozaki T. Lee H. Zou J. Saitoh T. (2013). Microtubule-driven spatial arrangement of mitochondria promotes activation of the NLRP3 inflammasome. *Nat. Immunol.* 14 454–460. 10.1038/ni.2550 23502856

[B45] Mohan V. Agarwal R. Singh R. P. (2016). A novel alkaloid, evodiamine causes nuclear localization of cytochrome-c and induces apoptosis independent of p53 in human lung cancer cells. *Biochem. Biophys. Res. Commun.* 477 1065–1071. 10.1016/j.bbrc.2016.07.037 27402273

[B46] Pan H. Xu L. H. Huang M. Y. Zha Q. B. Zhao G. X. Hou X. F. (2015). Piperine metabolically regulates peritoneal resident macrophages to potentiate their functions against bacterial infection. *Oncotarget* 6 32468–32483. 10.18632/oncotarget.5957 26439699 PMC4741706

[B47] Pearce L. V. Petukhov P. A. Szabo T. Kedei N. Bizik F. Kozikowski A. P. (2004). Evodiamine functions as an agonist for the vanilloid receptor TRPV1. *Org. Biomol. Chem.* 2 2281–2286. 10.1039/B404506H 15305207

[B48] Piccini A. Carta S. Tassi S. Lasiglie D. Fossati G. Rubartelli A. (2008). ATP is released by monocytes stimulated with pathogen-sensing receptor ligands and induces IL-1beta and IL-18 secretion in an autocrine way. *Proc. Natl. Acad. Sci. U.S.A.* 105 8067–8072. 10.1073/pnas.0709684105 18523012 PMC2430360

[B49] Prass K. Meisel C. Hoflich C. Braun J. Halle E. Wolf T. (2003). Stroke-induced immunodeficiency promotes spontaneous bacterial infections and is mediated by sympathetic activation reversal by poststroke T helper cell type 1-like immunostimulation. *J. Exp. Med.* 198 725–736. 10.1084/jem.20021098 12939340 PMC2194193

[B50] Py B. F. Jin M. Desai B. N. Penumaka A. Zhu H. Kober M. (2014). Caspase-11 controls interleukin-1beta release through degradation of TRPC1. *Cell Rep.* 6 1122–1128. 10.1016/j.celrep.2014.02.015 24630989 PMC4239700

[B51] Qiu C. Gao L. N. Yan K. Cui Y. L. Zhang Y. (2016). A promising antitumor activity of evodiamine incorporated in hydroxypropyl-beta-cyclodextrin: pro-apoptotic activity in human hepatoma HepG2 cells. *Chem. Cent. J.* 10:46. 10.1186/s13065-016-0191-y 27458481 PMC4959055

[B52] Saleh M. Mathison J. C. Wolinski M. K. Bensinger S. J. Fitzgerald P. Droin N. (2006). Enhanced bacterial clearance and sepsis resistance in caspase-12-deficient mice. *Nature* 440 1064–1068. 10.1038/nature04656 16625199

[B53] Shen P. Zhang Z. Zhu K. Cao H. Liu J. Lu X. (2019). Evodiamine prevents dextran sulfate sodium-induced murine experimental colitis via the regulation of NF-kappaB and NLRP3 inflammasome. *Biomed. Pharmacother.* 110 786–795. 10.1016/j.biopha.2018.12.033 30554117

[B54] Shi J. Zhao Y. Wang K. Shi X. Wang Y. Huang H. (2015). Cleavage of GSDMD by inflammatory caspases determines pyroptotic cell death. *Nature* 526 660–665. 10.1038/nature15514 26375003

[B55] Shoji N. Umeyama A. Takemoto T. Kajiwara A. Ohizumi Y. (1986). Isolation of evodiamine, a powerful cardiotonic principle, from Evodia rutaecarpa Bentham (Rutaceae). *J. Pharm. Sci.* 75 612–613. 10.1002/jps.2600750619 3735108

[B56] Song H. Liu B. Huai W. Yu Z. Wang W. Zhao J. (2016). The E3 ubiquitin ligase TRIM31 attenuates NLRP3 inflammasome activation by promoting proteasomal degradation of NLRP3. *Nat. Commun.* 7:13727. 10.1038/ncomms13727 27929086 PMC5155141

[B57] Wang L. Eftekhari P. Schachner D. Ignatova I. D. Palme V. Schilcher N. (2018). Novel interactomics approach identifies ABCA1 as direct target of evodiamine, which increases macrophage cholesterol efflux. *Sci. Rep.* 8:11061. 10.1038/s41598-018-29281-1 30038271 PMC6056500

[B58] Wegiel B. Larsen R. Gallo D. Chin B. Y. Harris C. Mannam P. (2014). Macrophages sense and kill bacteria through carbon monoxide-dependent inflammasome activation. *J. Clin. Invest.* 124 4926–4940. 10.1172/jci72853 25295542 PMC4347244

[B59] Wu W. S. Chien C. C. Liu K. H. Chen Y. C. Chiu W. T. (2017). Evodiamine prevents glioma growth, induces glioblastoma cell apoptosis and cell cycle arrest through JNK activation. *Am. J. Chin. Med.* 45 879–899. 10.1142/S0192415X17500471 28514905

[B60] Yang D. Li L. Qian S. Liu L. (2018). Evodiamine ameliorates liver fibrosis in rats via TGF-beta1/Smad signaling pathway. *J. Nat. Med.* 72 145–154. 10.1007/s11418-017-1122-5 28936800

[B61] Yang F. M. Zuo Y. Zhou W. Xia C. Hahm B. Sullivan M. (2018). sNASP inhibits TLR signaling to regulate immune response in sepsis. *J. Clin. Invest.* 128 2459–2472. 10.1172/jci95720 29733298 PMC5983344

[B62] Zantl N. Uebe A. Neumann B. Wagner H. Siewert J. R. Holzmann B. (1998). Essential role of gamma interferon in survival of colon ascendens stent peritonitis, a novel murine model of abdominal sepsis. *Infect. Immun.* 66 2300–2309. 9573121 10.1128/iai.66.5.2300-2309.1998 PMC108195

[B63] Zha Q. B. Wei H. X. Li C. G. Liang Y. D. Xu L. H. Bai W. J. (2016). ATP-induced inflammasome activation and pyroptosis is regulated by AMP-activated protein kinase in macrophages. *Front. Immunol.* 7:597. 10.3389/fimmu.2016.00597 28018360 PMC5149551

[B64] Zhou P. Li X. P. Jiang R. Chen Y. Lv X. T. Guo X. X. (2019). Evodiamine inhibits migration and invasion by Sirt1-mediated post-translational modulations in colorectal cancer. *Anticancer Drugs* 10.1097/CAD.0000000000000760 [Epub ahead of print]. 30789361 PMC6530977

