# Supplementary material for: Evodiamine Augments NLRP3 Inflammasome Activation and Anti-bacterial Responses Through Inducing α-Tubulin Acetylation
Source: Front Pharmacol. 2019 Mar 26;10:290. doi: 10.3389/fphar.2019.00290 (PMC6443907; doi:10.3389/fphar.2019.00290)
Supplement: Supplementary file 1 [file Data_Sheet_1.PDF]

Supplementary Information

Evodiamine augments NLRP3 inflammasome activation and anti-bacterial responses through inducing α-tubulin acetylation

(Chen-Guang Li[1†], Qiong-Zhen Zeng[1†], Ming-Ye Chen[1†], Li-Hui Xu[2], Cheng-Cheng Zhang[1], Feng-Yi Mai[1], Chen-Ying Zeng[1], Xian-Hui He[1*], Dong-Yun Ouyang[1*])

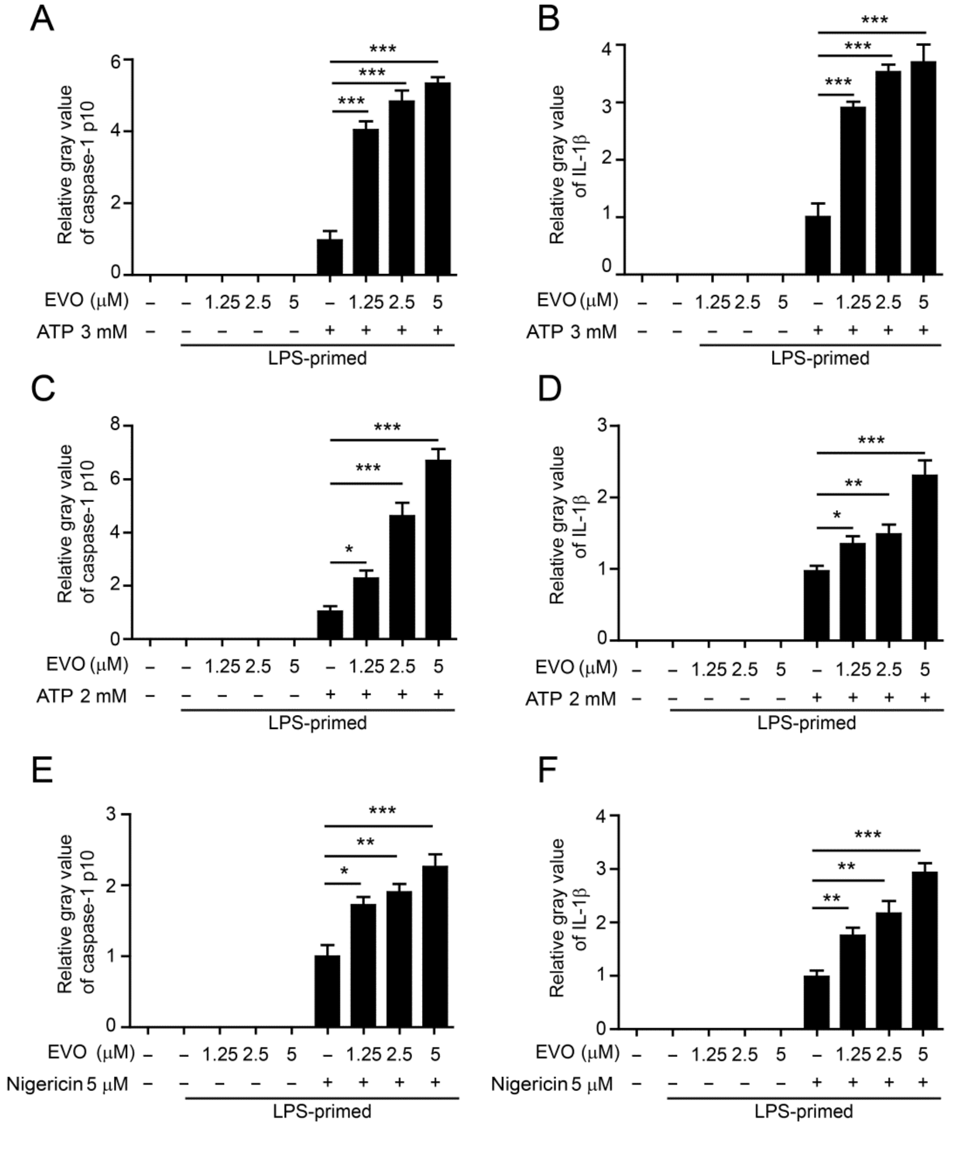

Figure S1 | Histograms showing the relative gray values of capase-1p10 or mature IL-1β levels in culture supernatants from J774A.1 cells and BMDMs. The blot images of Figure 1 A-C were captured by FluorChem8000 imaging system. The gray values of capase-1p10 and IL-1β bands were analyzed by AlphaEaseFC 4.0 software, and those values in ATP or nigericin group was set to 1.0 with those of the other groups being calculated relatively to the ATP or nigericin group, respectively (n = 3). Data are shown as mean ± SD *(n* = 5). (**A,B**) Data were corresponding to Figure 1**A**. (**C,D)** Data were corresponding to Fig. 1B. (**E,F**) were

corresponding to Fig. 1**C**. One-way analysis of variance (ANOVA): *P* < 0.0001 (A-D and F), *P* = 0.0003 (E); Tukey's *post hoc* test: **P* < 0.05, ***P* < 0.01, ****P* < 0.001. EVO, evodiamine.

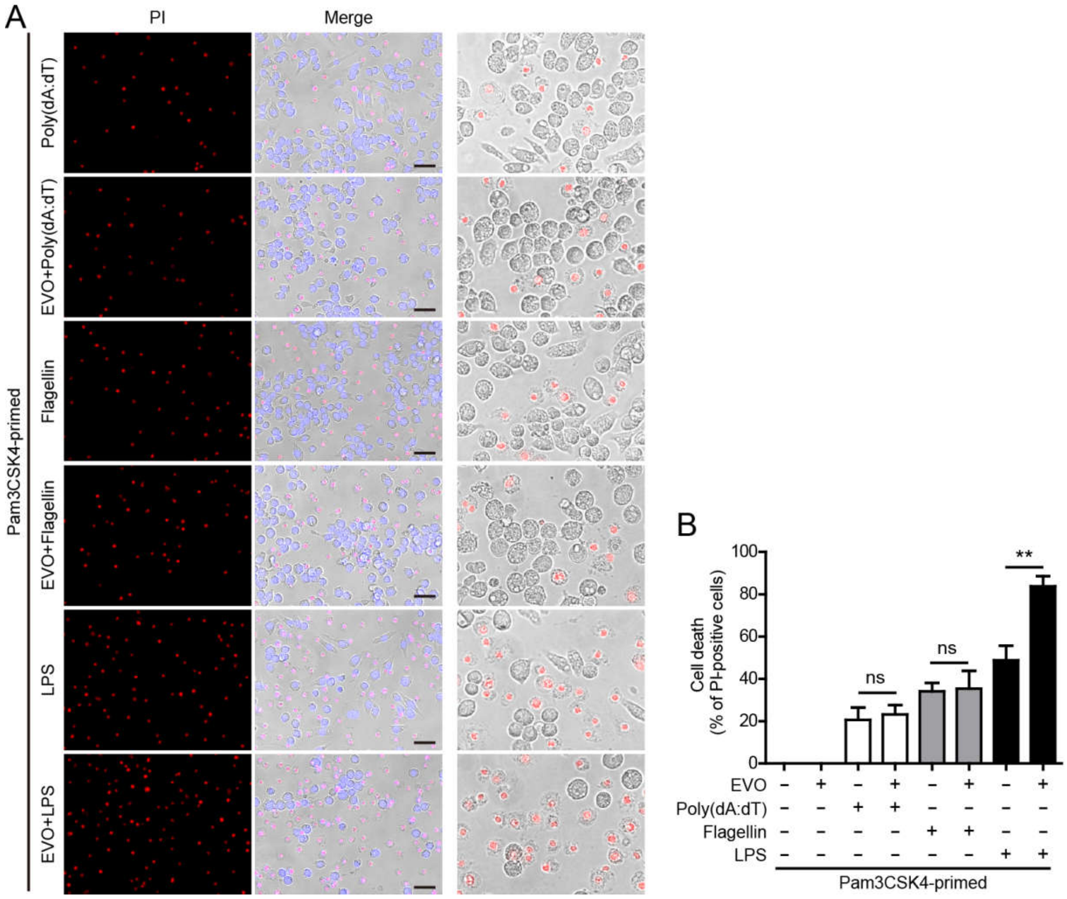

Figure S2 | Cell death induced by transfected with Poly(dA:dT), flagellin or LPS, and co-treatment with evodiamine in Pam3CSK4-primed J774A.1 cells. Cells were treated as in Figure 1H. (**A**) Cell death was measured by staining with propidium iodide (PI) (red, staining dead cells) and Hoechst 33342 (blue, staining total cells) together for 10 min. All images were captured by fluorescence microscopy and showed in merge with bright-field images. One set of representative images of three independent experiments are shown. Scale bars, 50 µm. (**B**) PI-positive cells were quantified by counting 5 randomly chosen fields (one field per well) containing around 100 cells each. Data are shown as mean ± SD (*n* = 5). Two-tailed Student's *t*-test: ***P* < 0.01; ns, not significant. EVO, evodiamine; Pam, Pam3CSK4.

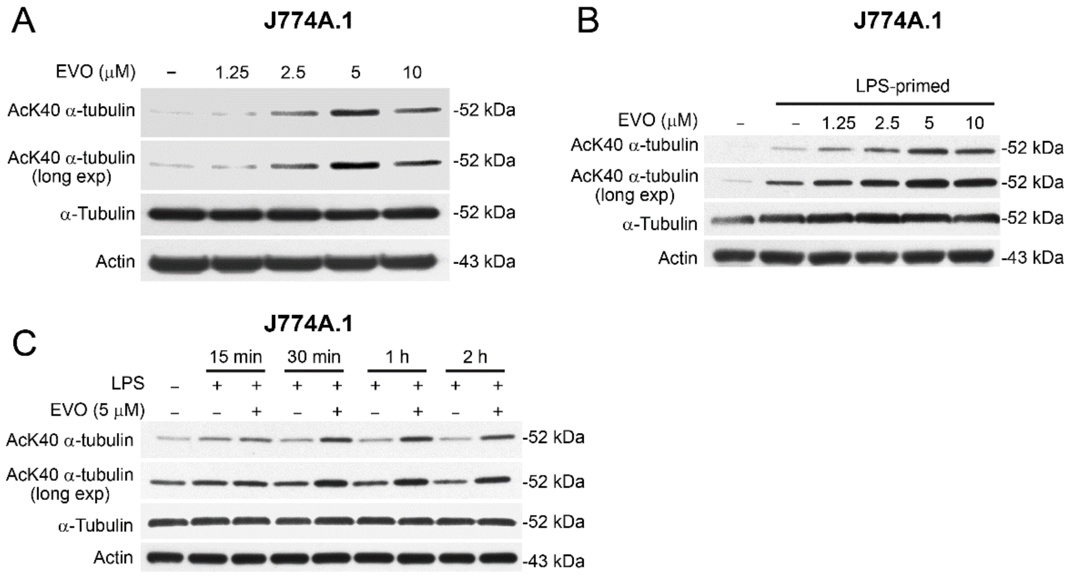

Figure S3 | Evodiamine induces acetylation of α-tubulin in J774A.1 cell. (A,B,C) J774A.1 cells were primed without (**A**) or with LPS (500 ng/ml) for 4 h, then treated with graded does of evodiamine for 1 h (**B**) or treated with evodiamine (5 μM) for indicated time periods (**C**) in the absence of LPS. Acetylated (K40) and total α-tubulin expression levels were determined by Western blotting. Actin was recruited as a loading control.

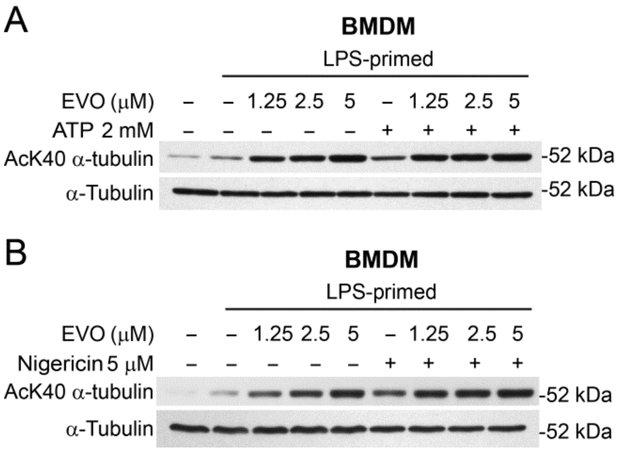

Figure S4 | Evodiamine elevated the expression of acetylated α-tubulin upon NLRP3 inflammasome activation in BMDMs. (A) Cells were treated in Figure 1B. (**B**) Cells were treated in Figure 1C. Acetylated and total α-tubulin expression levels were determined by Western blotting.

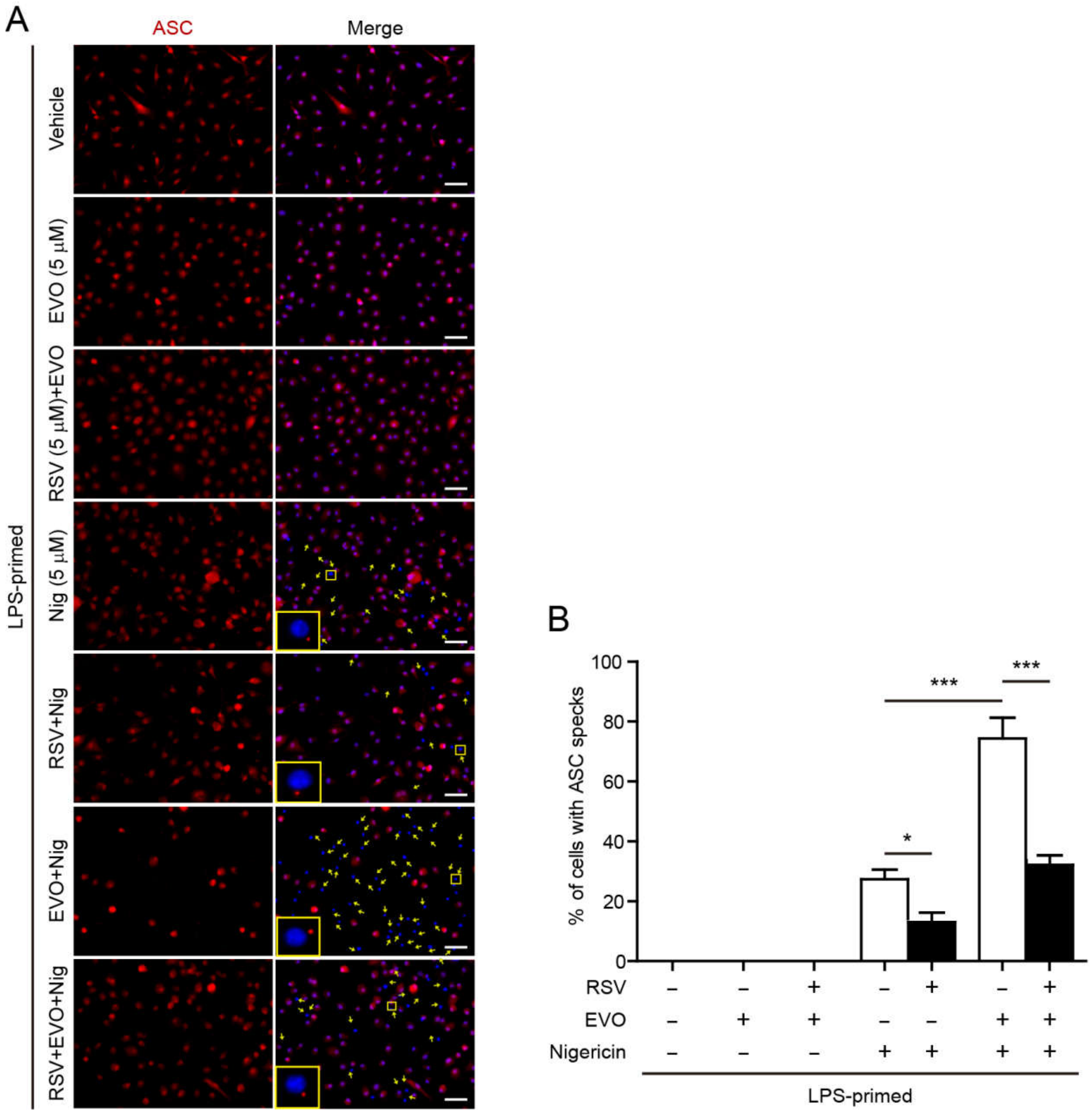

Figure S5 | Resveratrol treatment attenuated evodiamine-mediated enhancement of nigericin-induced ASC speck formation. (**A**) LPS-primed BMDMs were pre-treated with resveratrol (5 μM) for 30 min, and then incubated with indicated doses of evodiamine for 1 h, followed by stimulated with nigericin (5 μM) for 1 h. Representative immunofluorescence images showing ASC (red) subcellular distribution. Nuclei (blue) were revealed by Hoechst 33342. The images for ASC and nuclei were captured, respectively, and merged together. Yellow arrows indicate ASC specks and the enlarged inset showing cells with an ASC speck in each cell. Scale bars, 50 μm; RSV, resveratrol; EVO, evodiamine; Nig, nigericin. (**B**) Percentages of cells with an ASC speck relative to total number of cells from 5 random fields (one field per well) each containing ~50 cells. Data are shown as mean ± SD (n = 5). One-way analysis of variance (ANOVA): $P < 0.0001$; Tukey *post hoc* test: $^{*}P < 0.05$, $^{***}P < 0.001$.

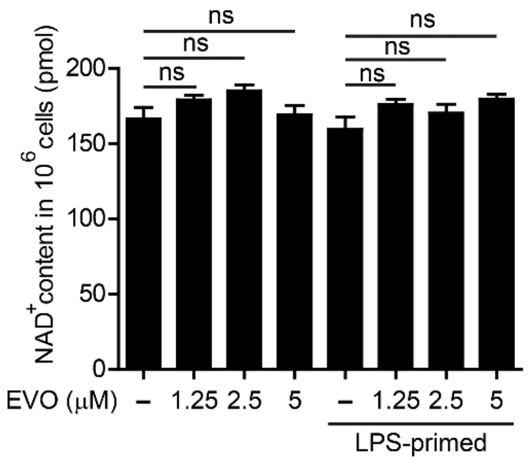

Figure S6 | Evodiamine treatment have no effects on intracellular NAD+ level in J774A.1 cell. J774A.1 cells were primed with or without LPS (500 ng/ml) for 4 h, then treated with graded does of evodiamine for 1 h. Intracellular NAD^+ levels were determined by NAD^+/NADH assay kit. Data are shown as mean ± SD (n = 3). One-way analysis of variance (ANOVA): P = 0.0175 (ns, not significant); Tukey's post hoc test: ns. EVO, evodiamine.

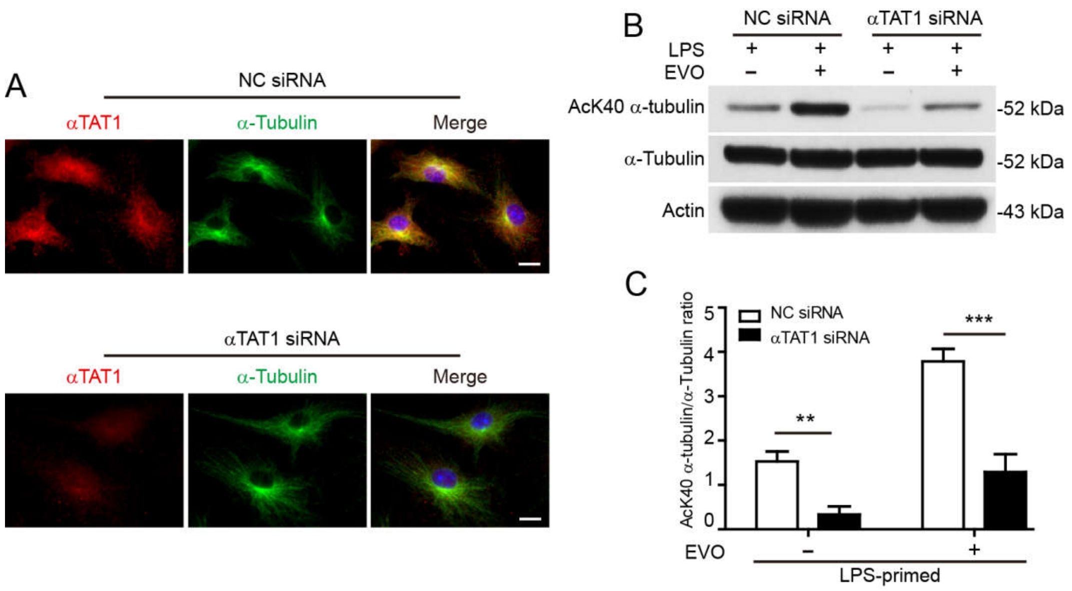

Figure S7 | Knockdown efficiency of αTAT1. (A,B) J774A.1 cells and BMDMs were transfected with negative control siRNA (NC siRNA) or *αTAT1*-specific siRNA (αTAT1 siRNA) for 72 h. (**A**) Representative immunofluorescence images showing the expression of αTAT1 (red) and α-tubulin (green) by knockdown of αTAT1 in BMDMs. Nuclei (blue) were stained with Hoechst 33342. Scale bars, 10 μm. (**B**) Cells were treated as in Figure 5C. Acetylated (K40) and total α-tubulin expression levels were determined by Western blotting. Actin was recruited as a loading control. The quantification of AcK40 α-tubulin relative to total α-tubulin in (**B**) is shown in (**C**) (n = 3). Two-tailed Student's t-test: **$P < 0.01$, ***$P < 0.001$. EVO, evodiamine.

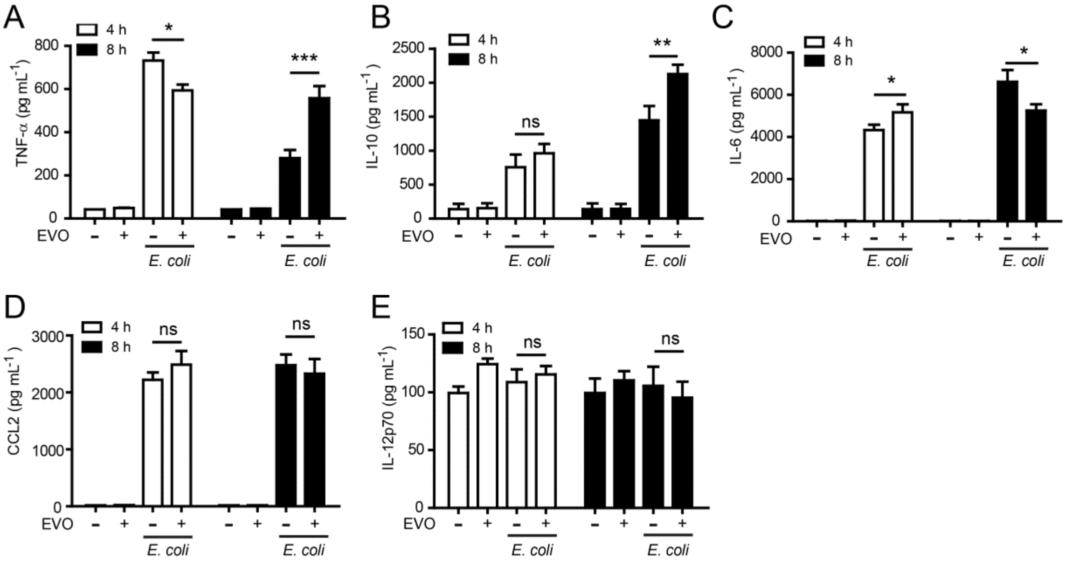

Figure S8 | Effect of evodiamine on the secretion of cytokines in the serum of mice infected with *E. coli.* Mice were treated as indicated in Figure 6E. Serum soluble cytokines TNF-α (**A**), IL-10 (**B**), IL-6 (**C**), CCL2 (**D**) and IL-12p70 (**E**) were measured by cytometric bead array (CBA) assay. The experiments were performed three times independently. Data are shown as mean ± SD (n = 5). One-way analysis of variance (ANOVA): $P < 0.0001$ (A-D), $P = 0.0025$ (E); Tukey *post hoc* test: *$P < 0.05$, **$P < 0.01$, ***$P < 0.001$, ns: not significant.

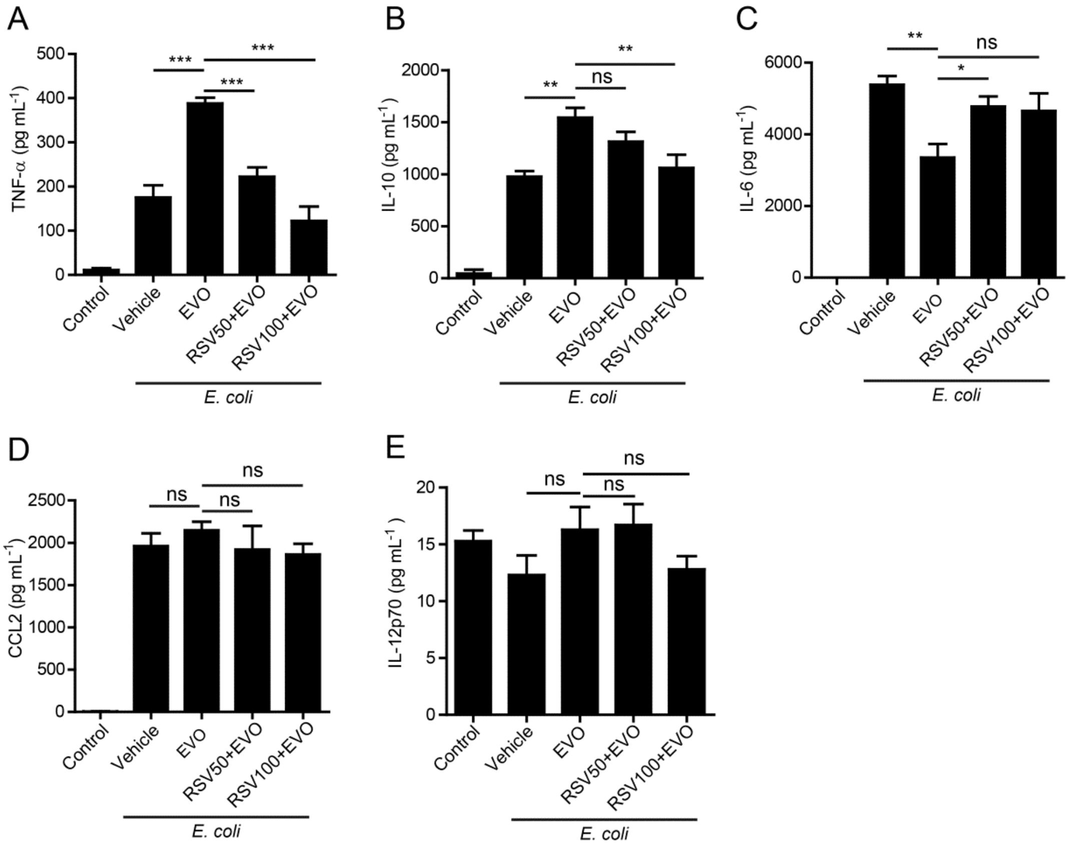

Figure S9 | Resveratrol pretreatment reversed evodiamine-mediated the secretion of cytokines in the serum of mice infected with *E. coli.* Mice were treated as indicated in Figure 7. Serum soluble cytokines TNF-α (**A**), IL-10 (**B**), IL-6 (**C**), CCL2 (**D**) and IL-12p70 (**E**) were measured by cytometric bead array (CBA) assay. The experiments were performed three times independently. Data are shown as mean ± SD (n = 5). One-way analysis of variance (ANOVA): $P < 0.0001$ (A-D), $P = 0.2806$ (E, not significant); Tukey *post hoc* test: *$P < 0.05$, **$P < 0.01$, ns: not significant.
